# Intake of dietary flavonoids in relation to overactive bladder among U.S. adults: a nutritional strategy for improving urinary health

**DOI:** 10.3389/fnut.2024.1437923

**Published:** 2024-07-24

**Authors:** Chaohuan Lin, Jie Lyu, Zhen Feng

**Affiliations:** ^1^Joint Centre of Translational Medicine, The First Affiliated Hospital of Wenzhou Medical University, Wenzhou, Zhejiang, China; ^2^Joint Centre of Translational Medicine, Wenzhou Institute, University of Chinese Academy of Sciences, Wenzhou, Zhejiang, China; ^3^Postgraduate Training Base Alliance of Wenzhou Medical University, Wenzhou, China; ^4^Oujiang Laboratory (Zhejiang Lab for Regenerative Medicine, Vision and Brain Health), Wenzhou, Zhejiang, China; ^5^College of Information and Engineering, Wenzhou Medical University, Wenzhou, Zhejiang, China

**Keywords:** overactive bladder, flavonoid, NHANES, cross-sectional study, nutrition

## Abstract

**Background:**

The increasing influence of overactive bladder (OAB) on physical as well as mental health of individuals is becoming more pronounced annually, as evidenced by the urge urinary incontinence and nocturia. Symptoms in OAB patients may be influenced by inflammation and oxidative stress. Flavonoids are recognized as significant anti-inflammatory and antioxidant agents, which are commonly available in fruits, tea, vegetables, etc. Previous research has demonstrated the therapeutic potential of flavonoids and their subclasses in treating inflammation, and oxidative stress. Despite this, there remains a paucity of research exploring the potential correlation between flavonoid consumption, specifically within distinct subclasses, and OAB. Thus, our study aims to investigate the relationship between flavonoid intake and OAB to identify possible dietary interventions for OAB management.

**Methods:**

We utilized the survey data from the National Health and Nutrition Examination Survey (NHANES) and the USDA Food and Nutrient Database for Dietary Studies (FNDDS) to investigate the relationship between dietary intake of total and subclass flavonoids and the risk of OAB based on 13,063 qualified American adults. The dietary flavonoid intake was estimated from two 24-h dietary recalls. Weighted multivariate logistic regression model, quantile-based g-computation, restricted cubic spline model, and stratified analysis were used to explore the association between flavonoid intake and OAB, respectively.

**Results:**

The participants diagnosed with OAB exhibited a higher percentage of being female, older, Non-Hispanic Black, unmarried, former drinkers, having a lower annual household income, lower poverty to income ratio, lower educational attainment, and a higher likelihood of being obese and smokers. Upon adjusting for confounding factors, the weighted logistic regression models revealed that the third quartile of consumption of anthocyanidin and the second quartile of consumption of flavone were significantly associated with the reduced odds of OAB, while total flavonoid consumption did not show a significant correlation with the risk of OAB. The quantile-based g-computation model indicated that flavone, anthocyanidin and flavonol were the primary contributors to the observed negative correlation. Furthermore, the restricted cubic spline models demonstrated a J-shaped non-linear exposure-response association between anthocyanidin intake and the risk of OAB (*P*_nonlinear_ = 0.00164). The stratified and interaction analyses revealed that the relationship between anthocyanidin intake and the risk of OAB was significantly influenced by age (*P*_interaction_ = 0.01) and education level (*P*_interaction_ = 0.01), while the relationship between flavone intake and the risk of OAB was found to vary by race (*P*_interaction_ = 0.02) and duration of physical activity (*P*_interaction_ = 0.05).

**Conclusion:**

Our research suggests that consuming a diet rich in flavonoid subclass anthocyanidin and flavone is associated with a reduced risk of OAB, potentially offering clinical significance in the prevention of OAB development. This underscores the importance of dietary adjustments in the management of OAB symptoms.

## 1 Introduction

As a clinical manifestation of Lower Urinary Tract Symptoms (LUTS), overactive bladder (OAB), is delineated by The International Continence Society (ICS) as a syndrome marked by urgent urination and night-time voiding, with or without urgent urinary incontinence (UUI), in the absence of urinary tract infection (UTI) or other discernible pathological conditions (1). As a complex of symptoms, OAB significantly impacts individual well-being and quality of life. Urinary incontinence can elicit feelings of embarrassment in individuals, potentially prompting withdrawal from social engagements and subsequent development of depressive symptoms (2). Nocturnal urination may disrupt sleep patterns, thereby contributing to heightened levels of anxiety and insomnia among affected patients (3, 4). A previous study has indicated that the collective prevalence of OAB in Asian nations like China and South Korea is estimated to be around 20.8% (5). A study conducted by the European Prospective Investigation into Cancer and Nutrition (EPIC) involving 19,165 participants from five European countries revealed an overall prevalence of 11.8% for OAB (6). Additionally, these findings consistently demonstrated a positive correlation between OAB prevalence and an increase in age. Therefore, there is a pressing need to enhance OAB prevention strategies and develop effective treatments, particularly in light of the rapidly aging global population. The pathophysiology of OAB is complex and currently not fully understood. Previous research has suggested that OAB can be linked to various factors, including obesity, diabetes, socioeconomic status, smoking, alcohol consumption (7, 8). Furthermore, symptoms in OAB patients may be influenced by inflammation, bacterial infections, and high levels of oxidative stress (9–12). Therefore, treatment for OAB may involve the use of anti-inflammatory or antioxidant medications, as well as antibiotics.

Flavonoids, recognized as significant anti-inflammatory and antioxidant agents, are commonly available in fruits, tea, vegetables, and cocoa (13). Classified as polyphenolic compounds, flavonoids can be categorized into six distinct classes: anthocyanidin, flavone, flavanone, flavan-3-ol, flavanol, and isoflavone (14). Many studies have demonstrated the therapeutic potential of flavonoids and their subclasses in treating inflammation, oxidative stress, and bacterial infections. Flavonoids were reported to harbor antioxidant ability by suppressing reactive oxygen species and free radicals in the body, thereby mitigating oxidative stress (13). Moreover, kaempferol, a specific subclass of flavonols, has been demonstrated to inhibit inflammatory responses in RA W264.7 cells stimulated by lipopolysaccharide through the suppression of inflammation-related genes via the NF-κB and MAPK signaling pathways (15). Research has demonstrated that certain flavonoid compounds, including diosmetin, baicalein, and silymarin, exhibit enhanced synergistic effects when combined with antimicrobial therapies, suggesting their potential as adjuvants for future antimicrobial treatments (16). Specifically, flavonoids can enhance the defense mechanisms of bacteria against antibiotics, including disruption of membrane function and DNA/RNA synthesis of bacteria. Given these findings, it is necessary to explore potential correlations between specific flavonoid subclasses and the risk of OAB. Until recently, there has been limited research on the association between flavonoid intake and OAB. Previous researches have primarily examined the potential therapeutic effects of flavonoid fractions on OAB (17, 18), without delving into the specific relationship between flavonoid subclasses and OAB. Given this, we propose to analyze the correlation between flavonoid intake and its subclasses with the incidence of overactive bladder by utilizing the National Health and Nutrition Examination Survey (NHANES) database.

The aim of this study is to utilize representative cohorts in the U.S. to evaluate the associations between the dietary consumption amounts of total and subclass flavonoids and the risk of OAB. Our study may provide valuable insights into dietary guidelines for individuals suffering from OAB from a nutritional standpoint.

## 2 Methods

### 2.1 Study population in this study

The National Health and Nutrition Examination Survey (NHANES) project is a prominent initiative to assess the health and nutritional well-being of both adults and children in the U.S. Each year, a nationally representative sample of around 5,000 participants is surveyed using a sophisticated multistage sampling approach, contingent upon the participants’ provision of written informed consent. The survey methodology comprises interviews encompassing demographics and dietary health, physical examinations involving medical and physiological assessments, and laboratory tests. Related contents are available at https://www.cdc.gov/nchs/nhanes/index.htm.

**Figure 1:**
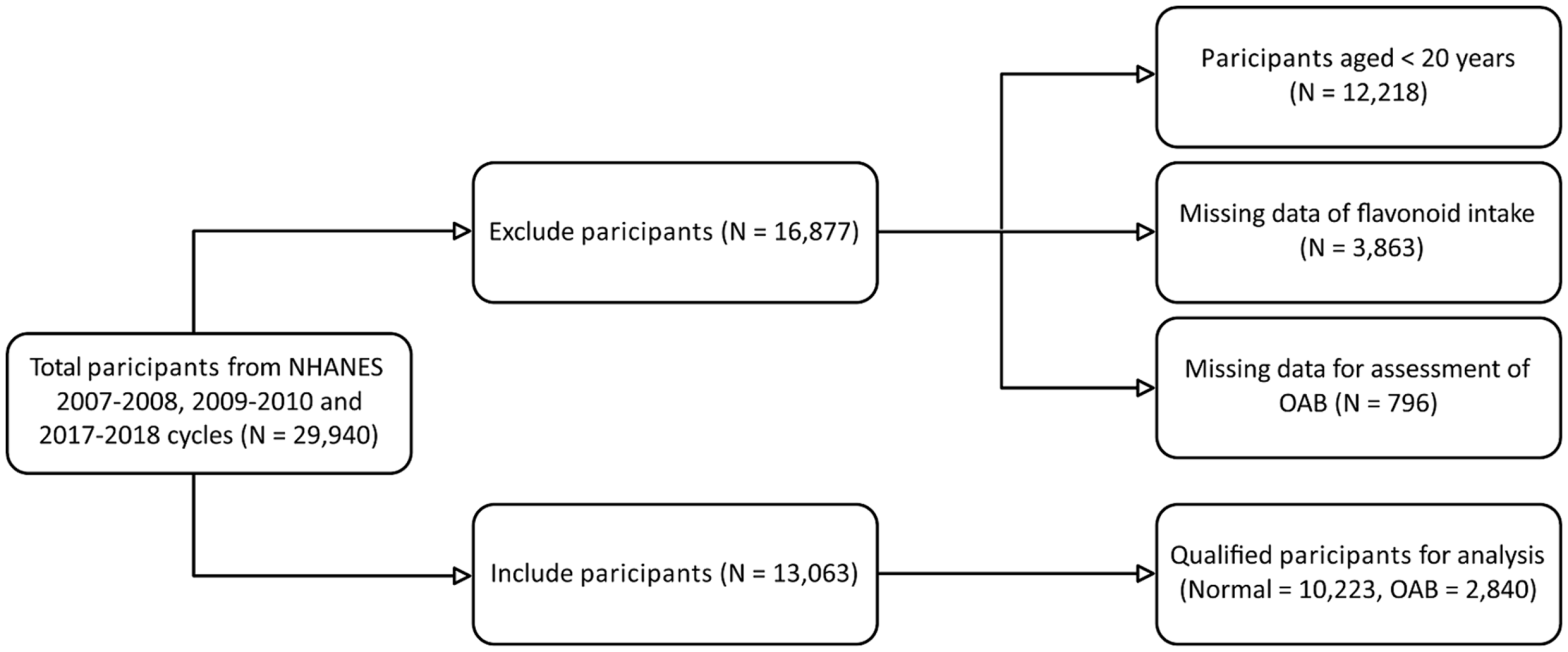
Flow diagram of participant selection from NHANES 2007–2010 and 2017–2018.

Figure 1, a flowchart, illustrates the initial enrolment of 29,940 participants from three NHANES cycles (2007–2008, 2009–2010, and 2017–2018) in our study. Subsequently, 16,877 participants were excluded due to criteria such as age younger than 20 years, missing data on intake of total flavonoid or its subclasses, and missing data on UUI and nocturia, which are essential for diagnosing OAB. Ultimately, a total of 13,063 participants were kept for further analysis.

### 2.2 Dietary flavonoid intake assessment

Information regarding the intake of total and subclass flavonoids can be accessed through the USDA Food and Nutrient Database for Dietary Studies (FNDDS), which includes data on the 29 flavonoid amounts (mg/100 g) of the six flavonoid subclasses found in all foods and beverages within the database (19). The intake data within the FNDDS is linked to additional diet and health-related information collected by “What We Eat in America” (WWEIA) or NHANES. Here, the data on dietary flavonoid intake for the years 2007–2008, 2009–2010, and 2017–2018 was analyzed in this study. The dietary flavonoid intake was estimated by averaging the data from two 24-h dietary recalls. One recall was conducted in person during a home interview, which gathered demographic and health information as well as dietary information from the previous 24 h. The second recall was conducted via telephone interview 3–10 days later.

### 2.3 Diagnosis of overactive bladder

Overactive bladder (OAB) should be viewed as a multifaceted syndrome rather than a singular disease, and therefore warrants comprehensive consideration when evaluating patients who exhibit common symptoms like nocturia and UUI. The NHANES database utilizes the Kidney Conditions – Urology Questionnaire to assess symptoms associated with OAB (20). Specifically, participants responded to the questions, “During the past 12 months, have you leaked or lost control of even a small amount of urine with an urge or pressure to urinate and you could not get to the toilet fast enough?” and “How frequently does this occur?,” the two questions were asked to evaluate the severity of UUI. The severity of nocturia is assessed by “During the past 30 days, how many times per night did you most typically get up to urinate, from the time you went to bed at night until the time you got up in the morning?”

In this work, the overactive bladder symptom score (OABSS) developed by Xiao et al. (21) was employed for the diagnosis of OAB. Xiao et al. utilized the responses to the aforementioned specific questions to quantify the severity of urinary incontinence and nocturia, resulting in assignment of scores. Diagnosis of OAB was based on the determination of whether the total OABSS exceeded a threshold of 3. The scoring criteria are demonstrated in Supplementary Figure 1.

### 2.4 Covariates

Through a comprehensive literature review, we have considered covariates that fall under two overarching categories: demographics and lifestyle. The demographic covariates encompass age, race/ethnicity, gender, educational attainment, poverty to income ratio (PIR), and marital status, while the lifestyle-related covariates consist of smoking habits, body mass index (BMI), alcohol consumption, daily caloric intake, score of Healthy Eating Index-2015 (HEI-2015), caffeine intake and duration of physical activity (PA).

The study population was categorized by age groups of 20–41, 42–61, and 62–80 years. Gender was dichotomized into male and female. Race was classified into five distinct groups: Non-Hispanic White, Mexican American, Non-Hispanic Black, Non-Hispanic Asian, and Other/Multi-Racial. Education level was divided into three distinct groups: less than high school, high school, and College and high. PIR was classified into three levels: <1, 1–3 and ≥ 3. Marital status was divided into two groups: married and not married. Smoking and alcohol status were classified as never, former and current. The concept of duration of PA encompasses the cumulative weekly duration of various activities such as walking or biking, household or yard tasks, muscle strengthening exercises, work-related PA, and recreational pursuits. BMI was categorized into three levels: <25, 25–30, and ≥ 30 kg/m^2^. Data on caffeine intake was calculated as the average of two 24-h recalls. The HEI-2015, which is informed by the Dietary Guidelines for Americans, was utilized to evaluate dietary quality (22). The total nutrient intake on the first day was computed to assess adherence to the HEI-2015 in this investigation.

### 2.5 Statistical analysis

In this study, all data analysis was done using R software (version 4.2.1), and the recommended sample weights sourced from the official NHANES website were incorporated into the weighted analyses. All statistical tests were conducted as two-sided. The significance level was set by *p*-value < 0.05.

The sampling weights were used in the weighted analyses according to the NHANES website. We obtained the 6-year sampling weight based on: 1/3 × dietary two-day 2-year sample weight (WTDR2D). The study population’s baseline characteristics were described using mean (standard deviation, SD) or median (interquartile range, IQR) for continuous variables and number (percentage, %) for categorical variables. To evaluate the statistical significance of two groups in continuous variables, the survey Student’s *t*-test was employed for assessing variables following normal distribution, while the survey Mann–Whitney Wilcoxon test was employed for assessing variables following non-normal distribution. The survey chi-square test was employed to calculate the statistical significance of categorical variables.

To explore the association between total flavonoid intake and its subclasses with the prevalence of OAB, the flavonoid intake data was stratified into four quartiles (quartile 1–4, Q1–4) as categorical variables and five weighted logistic regression models were constructed. Weighted multivariate logistic regression models were built based on the functions svyglm in the nhanesR package. We used odds ratios (ORs) together with 95% confidence intervals (CIs) to characterize the relationship between flavonoid subclass intakes and OAB risk. In the initial crude model, no confounding factor was adjusted. In model 1, sex was adjusted. In model 2, sex and age were adjusted. In model 3, sex, age and race were adjusted. In model 4, all covariates were adjusted (i.e., the complete model), including education level, marital status, poverty to income ratio (PIR), healthy eating index-2015 (HEI-2015), smoking status, total energy intake (kcal/day), alcohol status, duration of PA, caffeine intake and body mass index (BMI). *P*-trend value was determined by utilizing the median value of each quartile as a continuous variable in the weighted multivariate logistic regression model. We carried out quantile-based g-computation (qgcomp) analysis (23) by the qgcomp.noboot function in qgcomp R package to evaluate mixed impact of intake of six flavonoid subclasses on the risk of OAB. The qgcomp model is based on covariates in the complete model (model 4) to stratify each flavonoid subclass into quartiles and allocates a weighted index, either positive or negative, to evaluate the mixed impact of flavonoid subclasses. Furthermore, following the adjustment of the covariates in model 4, a model with restricted cubic splines (RCS) with three knots was used to analyze the expose-response associations and test for nonlinearity between the intake of subclasses of flavonoids and the risk of OAB in the study participants. Finally, a stratified and interaction analysis was conducted with consideration of all confounding factors included in model 4 to study the influence of subclasses of flavonoids on the risk of OAB.

## 3 Results

### 3.1 Baseline characteristics of the enrolled participants

Table 1 and Supplementary Table 1 display the baseline statistics of the study participants, categorized by the status of OAB. We encompassed a total of 13,063 participants from three two-year NHANES cycles, representing approximately 212.735 million U.S. residents. Of these participants, 2,840 were identified as having OAB based on previously defined diagnostic criteria (Supplementary Figure 1). As indicated in Table 1, the baseline table presents the demographic characteristics of participants with and without OAB. Those diagnosed with OAB exhibited a higher percentage of being female, older, Non-Hispanic Black, unmarried, former drinkers, having a lower annual household income (under $20,000), lower poverty to income ratio (PIR), lower educational attainment, and a higher likelihood of being obese (BMI ≥ 30 kg/m^2^) and smokers. Regarding disease related characteristics (Supplementary Table 1), individuals with OAB were found to have a higher prevalence of comorbidities such as diabetes mellitus (DM), hypertension, hyperlipidemia, cardiovascular disease (CVD), stroke and Parkinson’s disease. Additionally, OAB participants exhibited elevated levels of hypersensitive C-reactive protein (Hs-CRP), fasting plasma glucose (FPG), and blood urea nitrogen, and decreased levels of estimated glomerular filtration rate (eGFR), duration of PA, urine creatinine, and calcium (Supplementary Table 1). In relation to flavonoid intake, it was observed that the participants with OAB tended to have a lower intake of flavonoids and pro-inflammatory nutrients, as evidenced by a lower oxidative balance score (OBS) and a higher dietary inflammatory index (DII) in Supplementary Table 1. While the median intake of total flavonoids and all subclasses was lower in OAB participants compared to non-OAB participants (Table 1), only the intake of anthocyanidin, flavone, flavonol, and isoflavone was significant between OAB and non-OAB participants.

**Table 1:** The characteristics of the participants by Overactive Bladder (OAB) status, weighted.

Characteristics	Overall (*n* = 13,063)	Non-OAB (*n* = 10,223)	OAB (*n* = 2,840)	*p*-value
Demographic				
Age, *n* (%)				**<0.0001**
20–41	4,450 (39.88)	4,044 (44.42)	406 (17.39)	
42–61	4,509 (37.55)	3,536 (37.20)	973 (39.25)	
62–80	4,104 (22.57)	2,643 (18.38)	1,461 (43.36)	
Sex, *n* (%)				**<0.0001**
Male	6,306 (47.78)	5,161 (49.93)	1,145 (37.16)	
Female	6,757 (52.22)	5,062 (50.07)	1,695 (62.84)	
Race, *n* (%)				**<0.0001**
Non-Hispanic White	6,008 (67.61)	4,769 (68.29)	1,239 (64.27)	
Mexican American	2040 (8.40)	1,633 (8.64)	407 (7.18)	
Non-Hispanic Black	2,685 (11.27)	1917 (9.95)	768 (17.85)	
Non-Hispanic Asian	480 (2.07)	418 (2.20)	62 (1.41)	
Other/ Multi-Racial	1850 (10.65)	1,486 (10.92)	364 (9.29)	
Education level, *n* (%)				**<0.0001**
Less than high school	1,315 (4.82)	894 (3.94)	421 (9.16)	
High school	4,999 (35.58)	3,749 (34.01)	1,250 (43.53)	
College and high	6,735 (59.52)	5,569 (62.04)	1,166 (47.30)	
Marital status, *n* (%)				**0.003**
Not married	4,716 (34.47)	3,536 (33.75)	1,180 (38.07)	
Married	8,340 (65.51)	6,682 (66.25)	1,658 (61.93)	
Annual household income, *n* (%)				**<0.0001**
<$20,000	2,529 (13.30)	1748 (11.91)	781 (23.70)	
≥$20,000	9,889 (82.82)	7,993 (88.09)	1896 (76.30)	
Poverty to income ratio, *n* (%)				**<0.0001**
<1	2,265 (12.36)	1,666 (12.17)	599 (19.39)	
1–3	5,162 (32.61)	3,939 (34.31)	1,223 (39.85)	
≥3	4,435 (47.63)	3,718 (53.52)	717 (40.76)	
Weight status, *n* (%)				**<0.0001**
Normal (BMI < 25)	3,449 (28.40)	2,910 (30.33)	539 (19.74)	
Overweight (25 ≤ BMI < 30)	4,330 (32.16)	3,500 (32.88)	830 (29.66)	
Obese (BMI ≥ 30)	5,176 (38.86)	3,749 (36.79)	1,427 (50.60)	
Alcohol status, *n* (%)				**<0.0001**
Never	1,636 (9.73)	1,205 (9.66)	431 (13.97)	
Former	1824 (10.60)	1,297 (10.08)	527 (17.49)	
Current	8,751 (73.72)	7,161 (80.27)	1,590 (68.53)	
Smoking status, *n* (%)				**<0.0001**
Never	7,105 (55.92)	5,739 (57.67)	1,366 (47.27)	
Former	3,334 (24.93)	2,443 (23.70)	891 (31.04)	
Current	2,623 (19.14)	2041 (18.63)	582 (21.69)	
Caffeine intake (mg/day), *n* (%)				0.43
≤90	6,533 (43.44)	5,052 (43.22)	1,481 (44.55)	
>90	6,530 (56.56)	5,171 (56.78)	1,359 (55.45)	
Flavonoid intake (mg/day), median (IQR)				
Total flavonoid	72.96 (26.10, 261.96)	74.11 (26.62, 265.81)	69.44 (23.99, 251.64)	0.06
Flavanone	0.58 (0.07, 15.10)	0.61 (0.07, 14.81)	0.51 (0.05, 17.68)	0.56
Anthocyanidin	2.35 (0.11, 13.92)	2.43 (0.13, 14.26)	1.86 (0.05, 10.25)	**0.01**
Flavone	0.55 (0.20, 1.16)	0.57 (0.21, 1.18)	0.45 (0.15, 1.05)	**<0.001**
Flavonol	13.80 (7.44, 24.15)	13.96 (7.66, 24.45)	12.80 (6.78, 22.39)	**<0.001**
Isoflavone	0.01 (0.00, 0.10)	0.01 (0.00, 0.11)	0.01 (0.00, 0.06)	**<0.001**
Flavan-3-ol	18.15 (5.50, 202.83)	18.48 (5.70, 207.56)	17.37 (4.46, 188.55)	0.06

IQR, Interquartile range. Bold value refers to *p*-value less than 0.05.

### 3.2 No significant association between intake of total flavonoid and OAB

As shown in Table 1, we did not observe statistically significant difference in total flavonoid consumption between participants with OAB and those without OAB (*p*-value = 0.06). In Supplementary Table 2, we still do not find a significant difference in OAB prevalence across quartile groups of total flavonoid intake (*p*-value = 0.52). As presented in Supplementary Table 2, a significant and negative association exists between a higher level of total flavonoid intake and the estimated glomerular filtration rate (eGFR), urine creatinine levels, and dietary inflammatory index (DII), while a positive correlation was found with blood urea nitrogen levels, HEI-2015 total score, oxidative balance score (OBS), and composite dietary antioxidant index (CDAI). Subsequent quartile-based multivariate logistic regression analyses were performed to investigate the association between the intake of total flavonoid and the prevalence of OAB. The results shown in Table 2 indicated that no significant association was observed between total flavonoid intake and occurrence of OAB in the fully adjusted model (model 4).

**Table 2:** The multivariate logistic regression analysis results of the association between quartiles of total and subclass flavonoid intake and the risk of Overactive Bladder (OAB), weighted.

Variables	Quartile 1 (Q1)	Quartile 2 (Q2)	Quartile 3 (Q3)	Quartile 4 (Q4)	*p*-trend
Total flavonoid (Range, mg/day)	[0, 24.625]	(24.625, 64.82]	(64.82, 220.305]	(220.305, 6974.47]	
Crude model [OR (95% CI)]	Reference	0.91 (0.76, 1.08)	0.90 (0.73, 1.12)	0.89 (0.76, 1.03)	0.15
Model 1 [OR (95% CI)]	Reference	0.91 (0.77, 1.08)	0.91 (0.74, 1.12)	0.87 (0.75, 1.02)	0.11
Model 2 [OR (95% CI)]	Reference	**0.81 (0.67, 0.98)**	**0.74 (0.59, 0.93)**	**0.75 (0.63, 0.88)**	**<0.001**
Model 3 [OR (95% CI)]	Reference	**0.82 (0.67, 1.00)**	**0.75 (0.59, 0.96)**	**0.79 (0.67, 0.94)**	**0.01**
Model 4 [OR (95% CI)]	Reference	0.93 (0.68, 1.27)	1.10 (0.77, 1.57)	1.10 (0.85, 1.43)	0.23
Flavanone (Range, mg/day)	[0, 0.06]	(0.06, 0.62]	(0.62, 18.985]	(18.985, 590.625]	
Crude model [OR (95% CI)]	Reference	0.94 (0.77, 1.16)	**0.80 (0.66, 0.97)**	1.00 (0.83, 1.20)	0.52
Model 1 [OR (95% CI)]	Reference	0.93 (0.76, 1.15)	**0.76 (0.62, 0.92)**	1.00 (0.83, 1.20)	0.44
Model 2 [OR (95% CI)]	Reference	0.87 (0.70, 1.07)	**0.66 (0.54, 0.80)**	**0.82 (0.67, 0.99)**	**0.01**
Model 3 [OR (95% CI)]	Reference	0.90 (0.72, 1.12)	**0.68 (0.55, 0.84)**	**0.81 (0.66, 0.99)**	**0.01**
Model 4 [OR (95% CI)]	Reference	1.19 (0.86, 1.64)	0.95 (0.70, 1.29)	1.13 (0.83, 1.55)	0.79
Anthocyanidin (Range, mg/day)	[0, 0.12]	(0.12, 2.06]	(2.06, 11.07]	(11.07, 756.1]	
Crude model [OR (95% CI)]	Reference	0.90 (0.72, 1.12)	0.88 (0.74, 1.05)	**0.72 (0.58, 0.90)**	**0.003**
Model 1 [OR (95% CI)]	Reference	0.87 (0.69, 1.09)	0.86 (0.72, 1.03)	**0.67 (0.54, 0.85)**	**<0.001**
Model 2 [OR (95% CI)]	Reference	**0.75 (0.59, 0.97)**	**0.69 (0.56, 0.84)**	**0.48 (0.38, 0.62)**	**<0.0001**
Model 3 [OR (95% CI)]	Reference	**0.76 (0.59, 0.98)**	**0.70 (0.57, 0.85)**	**0.52 (0.40, 0.67)**	**<0.0001**
Model 4 [OR (95% CI)]	Reference	0.90 (0.64, 1.26)	**0.73 (0.55, 0.98)**	0.72 (0.49, 1.06)	**0.04**
Flavone (Range, mg/day)	[0, 0.185]	(0.185, 0.51]	(0.51, 1.09]	(1.09, 87.245]	
Crude model [OR (95% CI)]	Reference	**0.80 (0.68, 0.94)**	**0.77 (0.65, 0.93)**	**0.69 (0.56, 0.84)**	**0.001**
Model 1 [OR (95% CI)]	Reference	**0.77 (0.65, 0.91)**	**0.76 (0.63, 0.91)**	**0.69 (0.56, 0.84)**	**0.001**
Model 2 [OR (95% CI)]	Reference	**0.70 (0.59, 0.83)**	**0.67 (0.55, 0.81)**	**0.60 (0.49, 0.73)**	**<0.0001**
Model 3 [OR (95% CI)]	Reference	**0.71 (0.59, 0.84)**	**0.70 (0.57, 0.86)**	**0.64 (0.51, 0.80)**	**<0.001**
Model 4 [OR (95% CI)]	Reference	**0.70 (0.54, 0.90)**	0.83 (0.63, 1.09)	0.80 (0.62, 1.04)	0.26
Flavonol (Range, mg/day)	[0, 6.932]	(6.932, 12.745]	(12.745, 22.398]	(22.398, 332.035]	
Crude model [OR (95% CI)]	Reference	**0.84 (0.72, 0.99)**	**0.82 (0.68, 0.99)**	**0.72 (0.61, 0.85)**	**<0.001**
Model 1 [OR (95% CI)]	Reference	0.87 (0.74, 1.02)	0.85 (0.70, 1.02)	**0.77 (0.65, 0.91)**	**0.003**
Model 2 [OR (95% CI)]	Reference	**0.82 (0.69, 0.99)**	**0.80 (0.67, 0.96)**	**0.72 (0.60, 0.87)**	**<0.001**
Model 3 [OR (95% CI)]	Reference	0.84 (0.70, 1.01)	0.84 (0.70, 1.01)	**0.78 (0.64, 0.96)**	**0.02**
Model 4 [OR (95% CI)]	Reference	0.83 (0.61, 1.13)	1.06 (0.81, 1.38)	0.98 (0.74, 1.28)	0.62
Isoflavone (Range, mg/day)	[0, 0]	(0, 0.01]	(0.01, 0.075]	(0.075, 390.6]	
Crude model [OR (95% CI)]	Reference	**0.80 (0.66, 0.97)**	0.88 (0.75, 1.04)	**0.69 (0.59, 0.82)**	**<0.001**
Model 1 [OR (95% CI)]	Reference	**0.80 (0.65, 0.97)**	0.88 (0.75, 1.04)	**0.70 (0.59, 0.83)**	**<0.001**
Model 2 [OR (95% CI)]	Reference	**0.74 (0.59, 0.93)**	0.86 (0.73, 1.01)	**0.76 (0.64, 0.90)**	**0.004**
Model 3 [OR (95% CI)]	Reference	**0.74 (0.59, 0.93)**	0.87 (0.74, 1.02)	**0.78 (0.66, 0.93)**	**0.01**
Model 4 [OR (95% CI)]	Reference	0.91 (0.65, 1.27)	0.83 (0.66, 1.05)	0.94 (0.71, 1.24)	0.44
Flavan-3-ol (Range, mg/day)	[0, 4.975]	(4.975, 15.63]	(15.63, 157.757]	(157.757, 6724.88]	
Crude model [OR (95% CI)]	Reference	**0.77 (0.64, 0.93)**	0.87 (0.71, 1.07)	**0.84 (0.70, 1.00)**	0.12
Model 1 [OR (95% CI)]	Reference	**0.78 (0.65, 0.94)**	0.88 (0.72, 1.07)	**0.83 (0.69, 0.99)**	0.09
Model 2 [OR (95% CI)]	Reference	**0.70 (0.57, 0.86)**	**0.75 (0.60, 0.93)**	**0.72 (0.60, 0.88)**	**0.003**
Model 3 [OR (95% CI)]	Reference	**0.72 (0.59, 0.89)**	**0.77 (0.61, 0.97)**	**0.77 (0.63, 0.94)**	**0.02**
Model 4 [OR (95% CI)]	Reference	0.77 (0.57, 1.05)	1.09 (0.75, 1.59)	0.97 (0.73, 1.28)	0.56

OR, odds ratio; 95% CI, 95% confidence interval. Crude model: No covariates were adjusted. Model 1: Sex was adjusted. Model 2: Sex and age were adjusted. Model 3: Sex, age and race were adjusted. Model 4: Sex, age, race, education level, poverty to income ratio (PIR), marital status, healthy eating index-2015 (HEI-2015), total energy intake (kcal/day), smoking status, alcohol status, physical activity time, caffeine intake and body mass index (BMI) were adjusted. The bold values refer to *p*-value < 0.05 indicated significant statistical difference.

### 3.3 The association between intakes of flavonoid subclasses and overactive bladder

We utilized three models: weighted logistic regression, quantile-based g-computation (qgcomp), and a three-knot restricted cubic spline (RCS) model to examine the correlation between the intake of six flavonoid subclasses and the prevalence of OAB.

To investigate the relationship between the flavonoid intakes and the risk of OAB, we generated quartiles for the total and six subclass flavonoid intakes, respectively. The baseline characteristics of the total and subclass flavonoid quartiles were shown in Supplementary Tables 2–8. As indicated in Supplementary Table 2, the baseline table presents the demographic characteristics of participants in Q1-Q4 of total flavonoid intake. Those with highest total or five specific subclass flavonoid intake quartile (Quartile 4) exhibited a higher percentage of being middle-aged, having a higher annual household income (higher than or equal to $20,000), higher poverty to income ratio (PIR), and higher educational attainment (Supplementary Tables 2–6, 8).

The weighted multivariate logistic regression models revealed that the third quartile (Q3) of anthocyanidin intake of the participants exhibited a reduced risk of OAB (OR_Q3_[95% CI], 0.73[0.55, 0.98]) than the reference (Q1) in the fully adjusted logistic regression model (model 4) (Table 2). Additionally, a significant decreasing trend in odds ratio (OR) was observed across the quartile Q1 to Q4 (*p*-value for trend = 0.04). Similarly, the second quartile (Q2) of flavone intake exhibited a decreased risk (OR_Q2_[95% CI], 0.70[0.54, 0.90]) of OAB compared to the reference (Q1). However, in model 4, no significant negative relationship was uncovered between flavanone, flavonol, isoflavone, and flavan-3-ol intake and the risk of OAB (Table 2). Therefore, we only presented the results of the subsequent analyses except qgcomp model analysis for the anthocyanidin and flavone subclasses due to space limitations.

**Figure 2:**
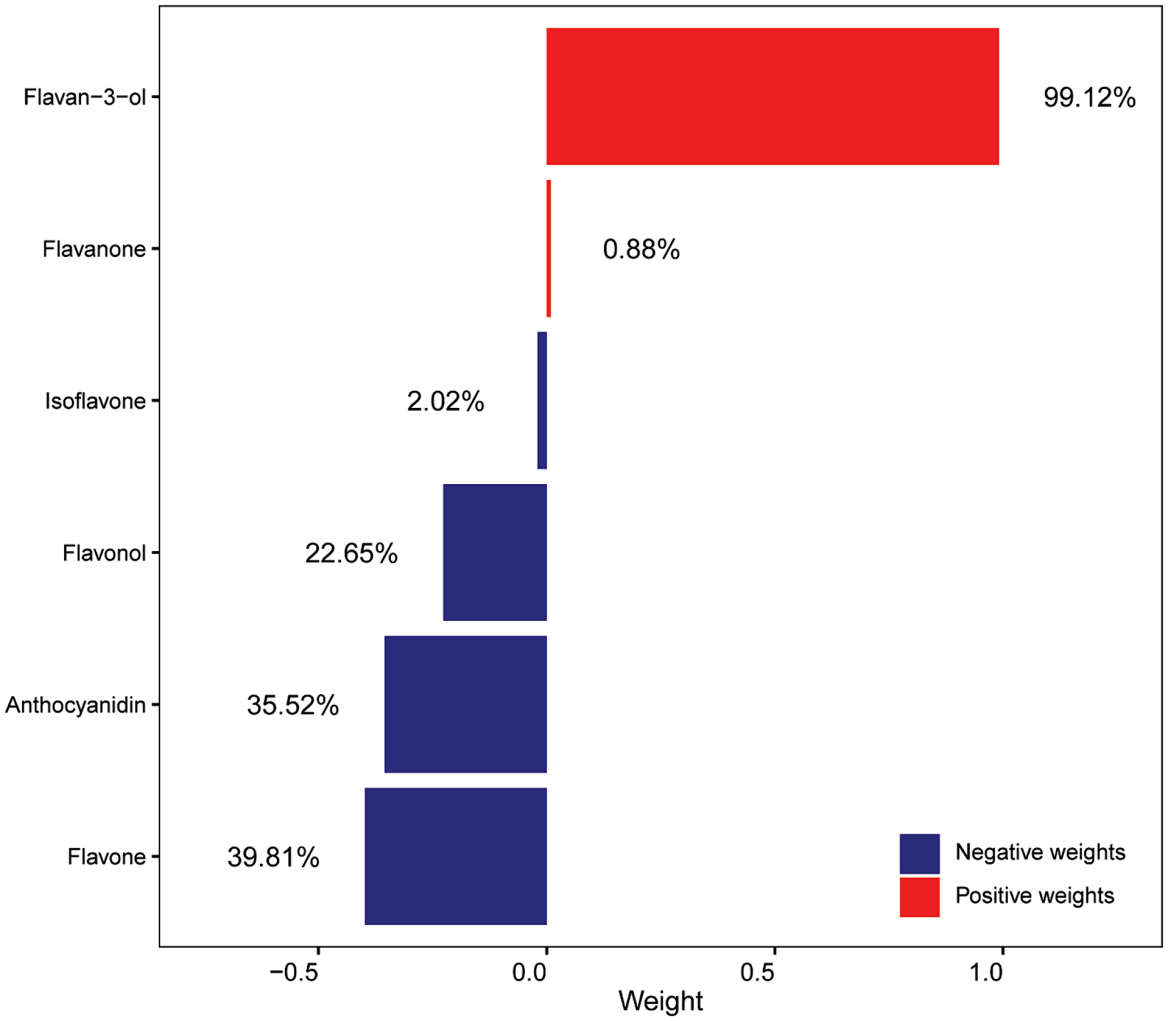
Weights of each flavonoid subclass scaled by quantile g-computation in the flavonoid mixture. All of the confounding factors that were used in the model 4 were adjusted.

Subsequently, the qgcomp model was utilized to assess the relationship between the combined effects of six flavonoid subclasses and the risk of OAB as well as to determine the estimated contribution of each dietary flavonoid subclass in the model (Figure 2). The result of qgcomp analysis indicated that the qgcomp index was not correlated with OAB risk (OR = 0.921, 95% CI [0.813, 1.043], *p*-value = 0.1986). Furthermore, the model effectively identified both positive and negative weights. Specifically, negative weights were associated with the intake of flavone, anthocyanidin, flavonol, and isoflavone, while positive weights were associated with the intake of flavan-3-ol and flavanone. Flavone accounted for the largest proportion of negative weights (39.81%), followed by anthocyanidin (35.52%), aligning with the findings of weighted logistic models.

**Figure 3:**
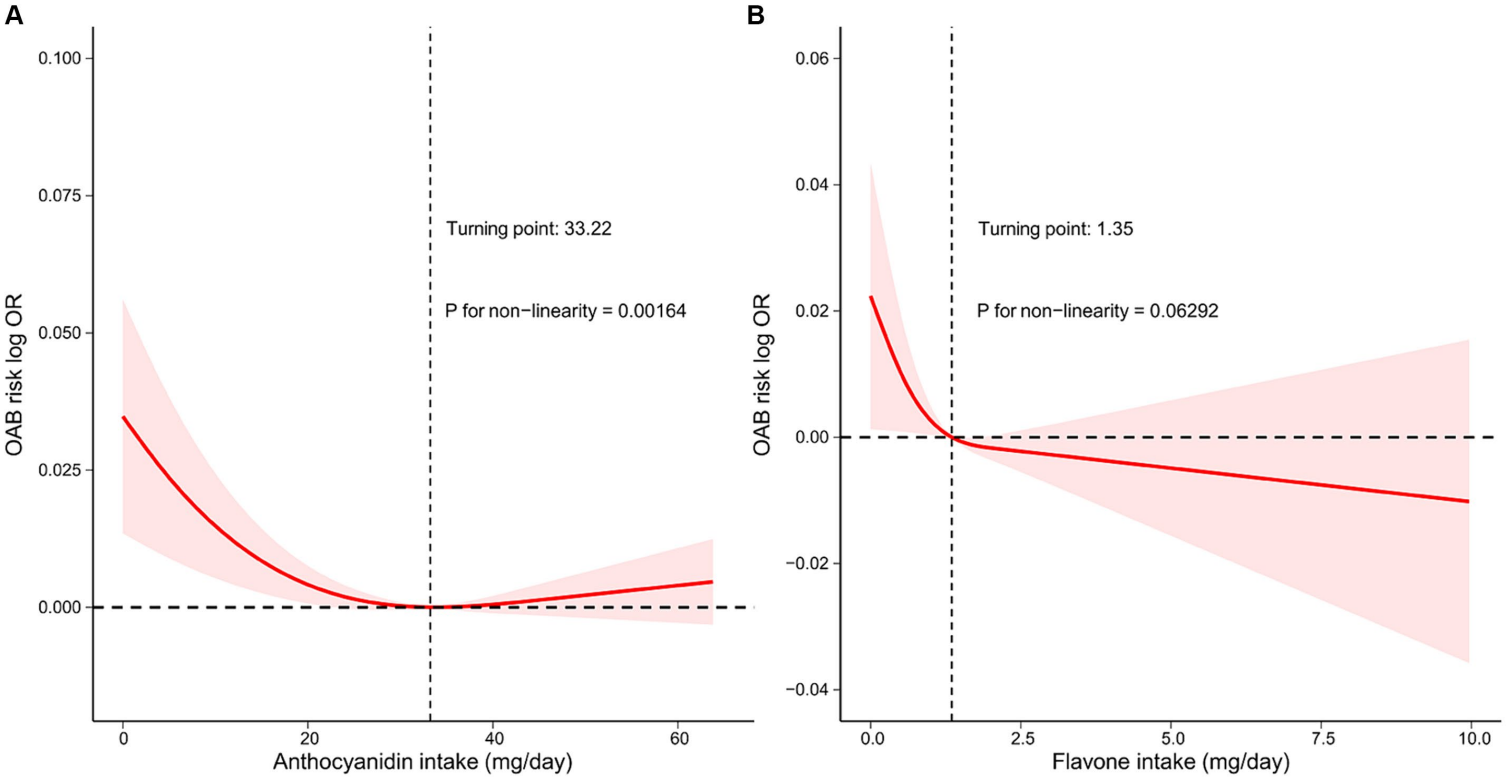
Restricted cubic spline model of the log OR of Overactive bladder (OAB) with intake amounts of two flavonoid subclasses, respectively, that is, **(A)** anthocyanidin and **(B)** flavone. All of the confounding factors that were used in the model 4 were adjusted. The red line represents a fitting spline. The red shading reflects 95% confidence interval. Dashed lines indicate turning points. Hazard ratio, HR; OR, Odds ratio.

Finally, we employed RCS analyses to examine the correlation between the intake of flavonoid subclasses and the risk of OAB. The findings, depicted in Figure 3A, indicate a J-shaped and negative exposure-response relationship between anthocyanidin intake and OAB risk (*P*_nonlinear_ = 0.00164). The negative relationship is limited to low amount of intake of anthocyanidin when daily anthocyanidin intake was below 33.22 mg per day. We found no significant evidence of nonlinearity (*P*_nonlinear_ = 0.06292) for the association between the intake of flavone and the risk of OAB (Figure 3B). However, the logarithm of the odds ratio (log OR) consistently decreased with increasing flavone intake, particularly when daily flavonoid intake was below 1.35 mg per day, indicating a stronger negative correlation.

Overall, our analyses demonstrated that a higher consumption of anthocyanidin and flavone were correlated with the reduced risk of OAB.

### 3.4 Stratified and interaction analyses

**Figure 4:**
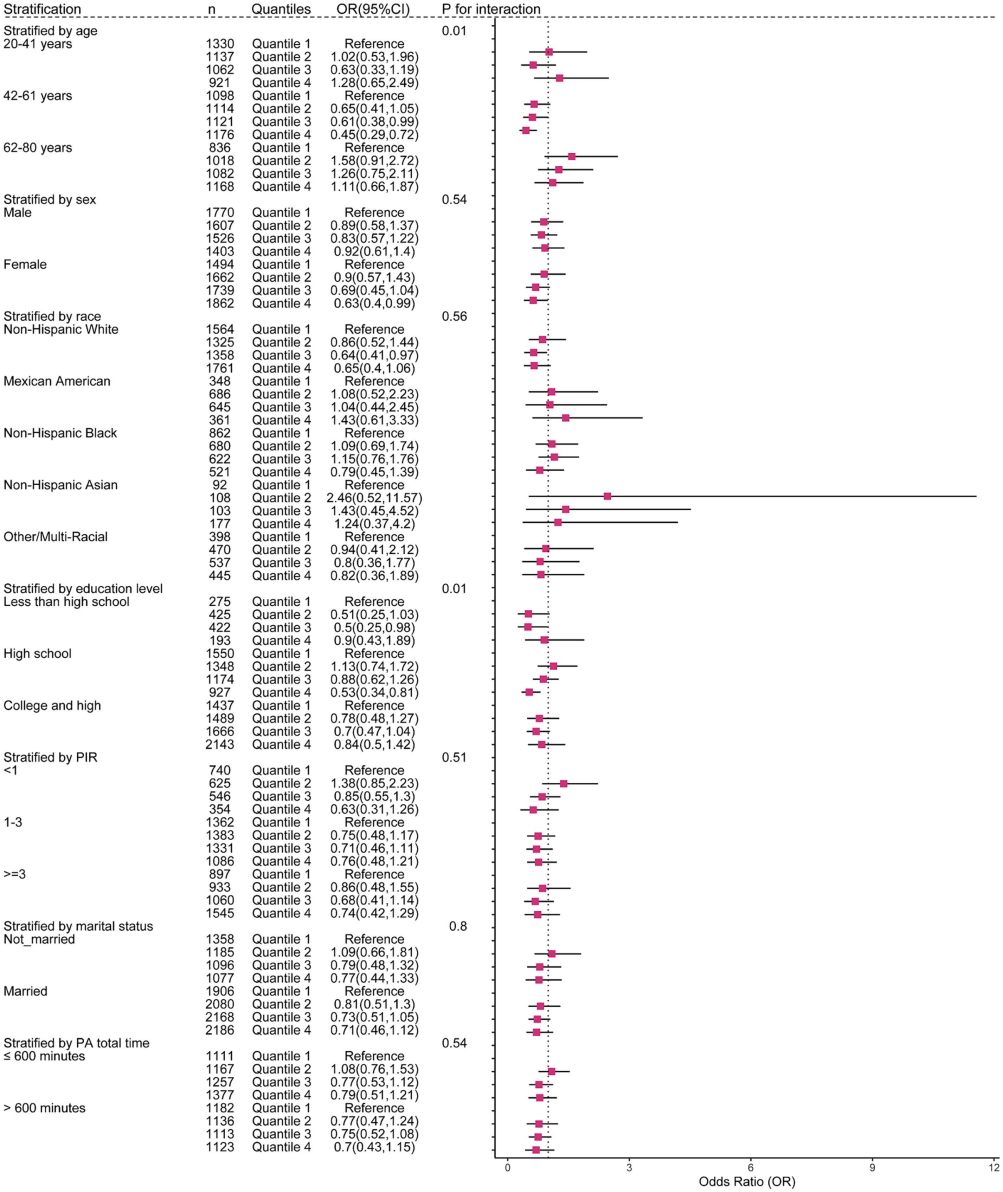
The weighted stratified and interaction analysis of association between the intake of anthocyanidin and confounding factors. All of the confounding factors present in model 4 are adjusted.

**Figure 5:**
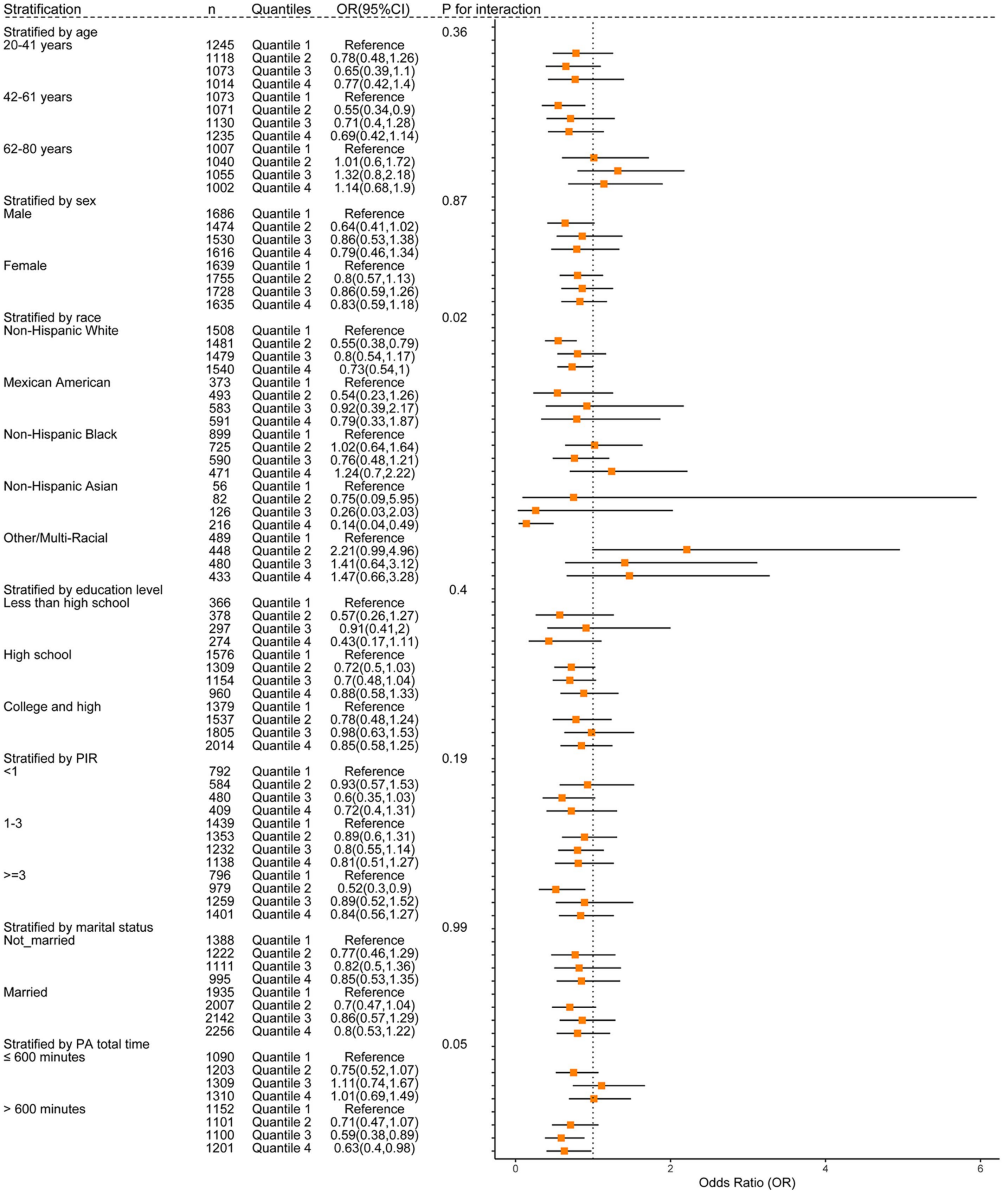
The weighted stratified and interaction analysis of association between the intake of flavone and confounding factors. All of the confounding factors present in model 4 are adjusted.

To obtain a better understanding of the association between anthocyanidin and flavone consumption and the risk of OAB, we further employed stratified and interaction analyses to evaluate if the association still holds when accounting for potential confounders. Upon controlling for the covariates used in model 4, except for the covariate that was used for stratification, we observed that the impact of anthocyanidin intake (Figure 4 and Supplementary Figure 2) on the risk of OAB was influenced by age (*P*_interaction_ = 0.01) and educational attainment (*P*_interaction_ = 0.01) groups. The association between intake of flavone and the risk of OAB (Figure 5 and Supplementary Figure 3) was found to be influenced by race (*P*_interaction_ = 0.02) and duration of physical activity (*P*_interaction_ = 0.05). Additionally, the risk of OAB decreased significantly with higher anthocyanidin intake (Q4) compared to baseline Q1 (Figure 4 and Supplementary Figure 2), in participants who were middle-aged (42–61 years old) (OR[95% CI], 0.45[0.29, 0.72]), female (0.63[0.4, 0.99]), had a high school education (0.53[0.34, 0.81]), a lower score on the HEI-2015 (0.5[0.27, 0.96]), and a BMI ranging from 25 to 30 kg/m^2^ (0.51[0.31, 0.86]). Participants who were self-reported as Non-Hispanic White (0.73[0.54, 1]) and Non-Hispanic Asian (0.14[0.04, 0.49]), engaged in higher duration of physical activity (0.63[0.4, 0.98]), had a higher energy intake (0.69[0.48, 0.99]), and maintained a normal BMI level (0.46[0.24, 0.91]), exhibited a significantly reduced risk of OAB when consuming higher flavone at Q4 levels compared to baseline Q1 levels (Figure 5 and Supplementary Figure 3).

## 4 Discussion

Overactive bladder (OAB) is a prevalent clinical symptom of the lower urinary tract that significantly affects the mental and physical health of patients, as well as contributing to a growing national economic burden (24). Hence, it is imperative to implement effective measures to mitigate the risk of OAB, with or without pharmacological intervention. A dietary management may be an efficient way to relieve the symptoms of OAB. In this cross-sectional study, we utilized weighted logistic regression analysis, restricted cubic spline (RCS), quantile g-computation (qgcomp) and stratified analysis to establish the relationship between dietary consumption of total and subclass flavonoids with the risk of OAB development. Table 3 presents a summary of results from the conducted model analyses in this study to help interpret the effects of flavonoid intake on OAB. We show that a higher dietary intake of anthocyanidins and flavones was significantly correlated with a reduced risk of OAB, whereas total flavonoid consumption was not significantly associated with OAB risk, implying only certain types of flavonoids may have a beneficial impact on alleviating OAB symptoms. Moreover, our study identified a notable expose-response correlation between anthocyanidin and flavone consumption and OAB. Stratified and interaction analyses revealed that increased intake of anthocyanidins seems to confer a more pronounced protective effect on middle-aged adults, women, individuals with a high school education level, those with a low HEI score, and overweight participants. Greater consumption of flavones was observed in non-Hispanic white and non-Hispanic Asian individuals, as well as in those with higher duration of PA, energy intake, and a normal BMI. Furthermore, a significant interaction between two variables, race and duration of physical activity, and flavone intake was found to impact the incidence of OAB. The association of anthocyanidin intake with the risk of OAB varies by age and education level. Overall, we suggest that the consumption of foods rich in anthocyanidins and flavones may be beneficial to lower the risk of OAB and this is especially true for specific populations.

**Table 3:** Summary of the effect of flavonoids on Overactive Bladder (OAB) by different models.

Model	Conclusion	Protective factor	Risk factor	Impact of flavonoid intake on OAB
Weighted multivariable logistic regression	There was no significant association between total flavonoid intake and OAB. Specific flavonoid intake (anthocyanidins and flavones) was significantly associated with a reduced risk of OAB	Anthocyanidin, Flavone	Total flavonoid, Flavonol, Flavan-3-ol, Flavanone, Isoflavone	Increased anthocyanidin intake (Q3) and moderate increases in flavone intake (Q2) were associated with reduced OAB risk
Quantile g-computation (qgcomp)	Flavone (39.81%), anthocyanidin (35.52%), flavonol (22.65%), and isoflavone (2.02%) intake were associated with a reduced risk of OAB	Anthocyanidin, Flavone, Flavonol, Isoflavone	Flavan-3-ol, Flavanone	Intake of flavonoid (especially anthocyanidins and flavones) showed significant protective effects in preventing OAB risk
Restricted Cubic Spline (RCS)	Anthocyanidin intake had a J-shaped negative expose-response relationship with OAB risk, and flavone intake had a linear negative correlation with OAB risk	Anthocyanidin, Flavone	NA	Moderate intake of foods containing anthocyanidins (below 33.22 mg/day) and flavones (below 1.35 mg/day) may have a potentially beneficial effect on reducing the risk of OAB
Weighted stratified and interaction analysis for anthocyanidin intake	Significant reduction in OAB risk with anthocyanidin intake among specific people. The impact was influenced by age and educational attainment groups	Middle-aged, Female, High school education level, Lower HEI-2015 score, Overweight BMI range	NA	Anthocyanidin intake significantly reduced the risk of OAB among middle-aged, female, high school educated, low HEI-2015 scores, and overweight individuals
Weighted stratified and interaction analysis for flavone intake	Significant reduction in OAB risk with flavone intake among specific people. The impact was influenced by race and duration of physical activity	Non-Hispanic White, Non-Hispanic Asian, Higher duration of physical activity, Higher energy intake, Normal BMI Range	NA	In Non-Hispanic White, Non-Hispanic Asian, people with higher duration of physical activity, higher energy intake and normal BMI, flavone intake was significantly associated with reduced risk of OAB

The pathophysiology of OAB remains incompletely understood due to its multifactorial etiologies. Prior research suggests that OAB may result from dysregulation of oxidative stress (12) and chronic inflammation within the body (25). A previous *in vitro* experiment showed that administering resveratrol, a polyphenol known for its antioxidant properties, effectively mitigated systemic and bladder oxidative stress in obese mice, resulting in significant improvements in cystometry alterations and bladder overactivity (26). A separate investigation demonstrated that the dysregulation of inflammatory proteins, especially IL-4 and TNF-α, may be implicated in the development of OAB (25). It is widely recognized that dietary flavonoids are crucial in modulating oxidative stress and exerting anti-inflammatory properties (27). Furthermore, the potential therapeutic effects of flavonoids in treating OAB might be attributed to the antioxidant and anti-inflammatory properties (28, 29). Our research indicates that greater consumption of flavonoids was associated with an increase in antioxidant levels, resulting in a reduction in an individual’s inflammatory potential (Supplementary Tables 2–4), as evidenced by a decrease in the DII index and an increase of the OBS and CDAI indexes. Nevertheless, our findings failed to reveal a statistically significant difference in total flavonoid intake among participants with OAB compared with those without OAB. Our study also indicated that the intake of total flavonoids does not act as an independent risk factor for OAB and distinct subclasses of flavonoids may elicit varying effects. Therefore, we focus on the analysis of two specific flavonoid subclasses in this study, given individual flavonoids within a specific flavonoid subclass typically demonstrate comparable biochemical impacts.

As a result, we indicate a significant inverse association between flavonoid subclasses and OAB. Specifically, the participants in the third anthocyanidin intake quartile showed a 27% decreased risk of OAB compared with the first anthocyanidin intake quartile (reference group) in the fully adjusted logistic regression model (Table 2). Additionally, the qgcomp model revealed that anthocyanidin intake represents the second highest weight of 35.52% among the six subclasses. The precise mechanisms by which anthocyanidins reduce the risk of OAB have not yet been fully elucidated. Anthocyanidins, a subgroup of flavonoids, are commonly present in human diets, such as berries and green vegetables. Due to the unique chemical structure, anthocyanidin has antioxidant, anti-inflammatory and neuroprotective attributes (30). In an *in vivo* study, the ingestion of blueberries rich in anthocyanidin was found to mitigate bladder dysfunction in rats afflicted with bladder outlet obstruction, thereby preventing the progress of OAB through its antioxidant properties and inhibition of bladder remodeling (31). Furthermore, studies have indicated that anthocyanidins possess estrogenic properties (32). A study conducted on animals demonstrated that anthocyanidin-rich compounds extracted from purple sweet potatoes could potentially be used as an alternative estrogen replacement therapy for the treatment of OAB (33). The primary mechanism involves phytoestrogens modulating gene expression through estrogen receptor binding, leading to the proliferation and hypertrophy of smooth muscle cells in bladder, ultimately enhancing their contractile strength and addressing OAB (33). Additionally, studies have indicated that dietary anthocyanins may improve insulin sensitivity (34). Research have illustrated a correlation between insulin resistance and OAB. In the bladder mucosa obese of mouse models, insulin resistance hindered the relaxation of the detrusor muscle, resulting in bladder overactivity (35, 36). Taken together, the potential impact of anthocyanidins on improving insulin resistance may have a significant role in mitigating the risk of OAB. Our research indicates that consuming approximately 33.22 mg of anthocyanidins daily is an effective way in preventing OAB. This amount is equivalent to consuming 332.2 g of strawberries or 4.3 g of bilberries per day (37), which is cost effective. In conclusion, our findings offer additional support for the positive effects of dietary anthocyanidin consumption on OAB.

Moreover, we indicated a negative association between the intake of flavone in the second quartile and the risk of OAB in the fully adjusted weighted logistic regression model. Additionally, the qgcomp model revealed that flavone intake represents the highest negative weight of 39.81% among the six subclasses. Flavones, such as apigenin and luteolin, are abundant in chamomile and parsley (38). Despite the scarcity of epidemiologic evidence on the relationship between flavones and OAB, our findings align with previous related studies. Flavoxate, a derivative of flavone, has been widely recognized for its approval in the treatment of OAB due to its superior efficacy and tolerability compared to other pharmaceutical agents (39). The primary mechanism of action of flavoxate involves inducing the relaxation of muscles through inhibiting L-type Ca^2+^ channels in the human detrusor muscle (40). In an *in vivo* experiment, rats were administered a diet supplemented with *Equisetum arvense* ethanol root extract, consisting primarily of apigenin and luteolin. This treatment resulted in a reduction in bladder activity by inhibiting the secretion of ATP from bladder epithelial cells and by scavenging free radicals to mitigate inflammation (29). Moreover, prior research has indicated that targeting TRPM8, a specific subtype of transient receptor potential (TRP) channels, may be a promising approach for managing OAB (41). Recent studies have validated the efficacy of flavone aglycones found in traditional herbal remedies as inhibitors of TRPM8 (42). Through antagonistic assays, the researchers illustrated the TRPM8 inhibitory effects of flavone aglycones found in traditional herbal extracts, suggesting potential clinical relevance due to the known benefits of TRPM8 inhibitors in treating symptoms of overactive bladder (42). However, further *in vivo* studies employing loss-of-function approaches, such as functional analysis in animal models and humans, are necessary to confirm the aforementioned conclusion. Therefore, consumption of dietary flavones may potentially alleviate symptoms of OAB through TRPM8 inhibition, a hypothesis that warrants further investigation. Collectively, our findings align with previous research indicating that sufficient consumption of flavones could serve as a moderately effective preventative measure for OAB.

Unexpectedly, our research failed to discover a negative correlation between flavonol intake and the risk of OAB. While the qgcomp results indicated a 22.65% negative correlation between flavonol intake and OAB, our logistic regression model did not reveal a significant negative association between any quartile of flavonol intake and OAB after controlling for all covariates under consideration. In contrast, research on kaempferol, a flavonol, has shown that it effectively inhibits lipopolysaccharide-induced ROS formation, mitigates oxidative stress induced by protamine sulfate, and reduces bladder irritation (28). Similarly, quercetin, a flavonol compound, has been shown to have a beneficial impact on the management of experimental overactive bladder through its effects on basophilic granulocyte function and the quantity of interstitial Cajal cells (43). The conflicting findings may be attributed to variations in the sample populations utilized, necessitating further research to confirm the potential of flavonol consumption in mitigating the risk of OAB.

To the best of our knowledge, our research is the initial investigation utilizing a nationally representative sample to systematically analyze the correlation between intake of total and subclass flavonoids and the risk of OAB. Moreover, the rigorous adjustment for numerous confounding factors and the utilization of stratified analyses serve to bolster the reliability of our findings. Altogether, our findings can serve as a foundation for future research on the impact of distinct flavonoid subclasses on the symptom relief of OAB. Further clinical trials are imperative to corroborate these results and elucidate the potential mechanisms by which anthocyanidins and flavones could mitigate the likelihood of OAB. Nevertheless, it is necessary to disclose the limitations present in our study. This study, as a cross-sectional survey, is unable to establish causal relationships. Additional large-scale randomized controlled trials are necessary to determine the causal relationship between flavonoid consumption and the risk of OAB, as well as to identify the safe dosage levels of flavonoids for various populations. Additionally, the evaluation of flavonoid intake data using two 24-h dietary recalls may not accurately reflect actual intake habits. Furthermore, the NHANES project does not include laboratory tests for OAB diagnosis, rather, OAB diagnosis is based exclusively on the Overactive Bladder Symptom Score (OABSS). However, the score depends on the symptoms obtained from questionnaires, potentially leading to recall bias. Lastly, the limited scope of NHANES data to the U.S. participants may restrict the generalizability of the main results to other countries.

## 5 Conclusion

In summary, weighted multivariate logistic regression model, restricted cubic spline model, and stratified analysis suggest a potential inverse relationship between increased anthocyanidin and flavone consumption and the incidence of overactive bladder. The quantile-based g-computation analysis indicates that flavone, anthocyanidin, and flavonol contribute more to the observed negative correlation than other flavonoid subclasses. Furthermore, the restricted cubic spline models demonstrated a J-shaped non-linear exposure-response relationship between anthocyanidin intake and OAB risk. The findings from the stratified and interaction analyses suggest that the relationship between anthocyanidin intake and OAB risk was significantly influenced by age and education level, while the association between flavone intake and OAB risk is influenced by race and duration of physical activity. These results imply that including foods rich in anthocyanidin and flavone in one’s diet may offer benefits in the prevention of OAB. Nevertheless, additional *in vitro* and clinical studies are necessary to further validate these results because of the cross-sectional nature of our study.

## Data availability statement

Publicly available datasets were analyzed in this study. This data can be found at: https://wwwn.cdc.gov/nchs/nhanes/.

## Ethics statement

The study of NHANES was approved by the National Centre for Health Statistics’ research ethics review board. All of the participants have already provided written informed consent and the study procedures. This study was exempt from human subject ethical review because the data we used can be freely obtained from the NHANES website.

## Author contributions

CL: Writing – original draft, Writing – review & editing, Data curation. JL: Writing – original draft, Writing – review & editing, Methodology, Data curation, Conceptualization. ZF: Writing – original draft.

## References

[1] Wein AJ Rovner ES. Definition and epidemiology of overactive bladder. Urology. (2002) 60:7–12. doi: 10.1016/s0090-4295(02)01784-3 12493342

[2] Lai HH Shen B Rawal A Vetter J. The relationship between depression and overactive bladder/urinary incontinence symptoms in the clinical OAB population. BMC Urol. (2016) 16:60. doi: 10.1186/s12894-016-0179-x, PMID: 27716241 PMC5053341

[3] Lai HH Rawal A Shen B Vetter J. The relationship between anxiety and overactive bladder or urinary incontinence symptoms in the clinical population. Urology. (2016) 98:50–7. doi: 10.1016/j.urology.2016.07.013, PMID: 27450939 PMC5116264

[4] Lu Z Zhang J Lin S Fan Z He Z Tang F. Associations between overactive bladder and sleep patterns: a cross-sectional study based on 2007–2014 NHANES. BMC Urol. (2023) 23:184. doi: 10.1186/s12894-023-01329-z, PMID: 37957629 PMC10642019

[5] Chuang YC Liu SP Lee KS Liao L Wang J Yoo TK. Prevalence of overactive bladder in China, Taiwan and South Korea: results from a cross-sectional, population-based study. Low Urin Tract Symptoms. (2017) 11:48–55. doi: 10.1111/luts.12193, PMID: 28967230 PMC7379992

[6] Irwin DE Milsom I Hunskaar S Reilly K Kopp Z Herschorn S. Population-based survey of urinary incontinence, overactive bladder, and other lower urinary tract symptoms in five countries: results of the EPIC study. Eur Urol. (2006) 50:1306–15. doi: 10.1016/j.eururo.2006.09.019, PMID: 17049716

[7] Wang Y Xu K Hu H Zhang X Wang X Na Y. Prevalence, risk factors, and impact on health related quality of life of overactive bladder in China. Neurourol Urodyn. (2011) 30:1448–55. doi: 10.1002/nau.21072, PMID: 21826714

[8] McKellar K Bellin E Schoenbaum E Abraham N. Prevalence, risk factors, and treatment for overactive bladder in a racially diverse population. Urology. (2019) 126:70–5. doi: 10.1016/j.urology.2018.12.021 30597170

[9] Brierley SM Goh KGK Sullivan MJ Moore KH Ulett GC Grundy L. Innate immune response to bacterial urinary tract infection sensitises high-threshold bladder afferents and recruits silent nociceptors. Pain. (2020) 161:202–10. doi: 10.1097/j.pain.0000000000001692, PMID: 31479069

[10] Montalbetti N Dalghi MG Bastacky SI Clayton DR Ruiz WG Apodaca G. Bladder infection with uropathogenic *Escherichia coli* increases the excitability of afferent neurons. Am J Physiol Renal Physiol. (2022) 322:F1–F13. doi: 10.1152/ajprenal.00167.2021, PMID: 34779263 PMC8698541

[11] Masuda H Kihara K Saito K Matsuoka Y Yoshida S Chancellor MB. Reactive oxygen species mediate detrusor overactivity via sensitization of afferent pathway in the bladder of anaesthetized rats. BJU Int. (2007) 101:775–80. doi: 10.1111/j.1464-410X.2007.07310.x, PMID: 18005207

[12] Wu Y-H Chueh K-S Chuang S-M Long C-Y Lu J-H Juan Y-S. Bladder hyperactivity induced by oxidative stress and bladder ischemia: a review of treatment strategies with antioxidants. Int J Mol Sci. (2021) 22:22. doi: 10.3390/ijms22116014, PMID: 34199527 PMC8199707

[13] Ahmed M Eun J-B. Flavonoids in fruits and vegetables after thermal and nonthermal processing: a review. Crit Rev Food Sci Nutr. (2017) 58:3159–88. doi: 10.1080/10408398.2017.1353480, PMID: 29035571

[14] Esposito S Gialluisi A Costanzo S Di Castelnuovo A Ruggiero E De Curtis A. Dietary polyphenol intake is associated with biological aging, a novel predictor of cardiovascular disease: cross-sectional findings from the Moli-Sani study. Nutrients. (2021) 13:13. doi: 10.3390/nu13051701, PMID: 34067821 PMC8157169

[15] Hwang D Kang MJ Kang CW Kim GD. Kaempferol-3-O-β-rutinoside suppresses the inflammatory responses in lipopolysaccharide-stimulated RAW264.7 cells via the NF-κB and MAPK pathways. Int J Mol Med. (2019) 44:2321–8. doi: 10.3892/ijmm.2019.4381, PMID: 31661129

[16] Malczak I Gajda A. Interactions of naturally occurring compounds with antimicrobials. J Pharm Anal. (2023) 13:1452–70. doi: 10.1016/j.jpha.2023.09.014, PMID: 38223447 PMC10785267

[17] Juan YS Chuang SM Lee YL Long CY Wu TH Chang WC. Green tea catechins decrease oxidative stress in surgical menopause-induced overactive bladder in a rat model. BJU Int. (2012) 110:E236-44. doi: 10.1111/j.1464-410X.2012.11258.x 22639915

[18] Fürer K Eberli D Betschart C Brenneisen R De Mieri M Hamburger M. Inhibition of porcine detrusor contractility by the flavonoid fraction of *Bryophyllum pinnatum* – a potential phytotherapeutic drug for the treatment of the overactive bladder syndrome. Phytomedicine. (2015) 22:158–64. doi: 10.1016/j.phymed.2014.11.009, PMID: 25636885

[19] FNDDS Flavonoid database: USDA ARS. Available at: https://www.ars.usda.gov/northeast-area/beltsville-md-bhnrc/beltsville-human-nutrition-research-center/food-surveys-research-group/docs/fndds-flavonoid-database/ (Accessed May 21, 2024).

[20] Kidney conditions – urology questionnaire of NHANES. Available at: https://wwwn.cdc.gov/Nchs/Nhanes/2017-2018/KIQ_U_J.htm (Accessed May 21, 2024).

[21] Xiao Y Yin S Wang J Cui J Yang Z Wang J. A positive association between the prevalence of circadian syndrome and overactive bladder in United States adults. Front Public Health. (2023) 11:1137191. doi: 10.3389/fpubh.2023.1137191, PMID: 37637821 PMC10449362

[22] Fan Y Ni S Zhang H. Association between healthy eating Index-2015 total and component food scores with osteoporosis in middle-aged and older Americans: a cross-sectional study with U.S. National Health and Nutrition Examination Survey. Osteoporosis Int. (2021) 33:921–9. doi: 10.1007/s00198-021-06247-0, PMID: 34854956

[23] Keil AP Buckley JP O’Brien KM Ferguson KK Zhao S White AJ. A quantile-based g-computation approach to addressing the effects of exposure mixtures. Environ Health Perspect. (2020) 128:47004. doi: 10.1289/ehp5838, PMID: 32255670 PMC7228100

[24] Coyne KS Wein A Nicholson S Kvasz M Chen C-I Milsom I. Economic burden of urgency urinary incontinence in the United States: a systematic review. J Manag Care Pharm. (2014) 20:130–40. doi: 10.18553/jmcp.2014.20.2.130, PMID: 24456314 PMC10437639

[25] Ma E Vetter J Bliss L Lai HH Mysorekar IU Jain S. A multiplexed analysis approach identifies new association of inflammatory proteins in patients with overactive bladder. Am J Physiol Renal Physiol. (2016) 311:F28–34. doi: 10.1152/ajprenal.00580.2015, PMID: 27029431 PMC4967156

[26] Alexandre EC Calmasini FB de Oliveira MG Silva FH da Silva CPV André DM. Chronic treatment with resveratrol improves overactive bladder in obese mice via antioxidant activity. Eur J Pharmacol. (2016) 788:29–36. doi: 10.1016/j.ejphar.2016.06.017, PMID: 27316789

[27] Chen G-L Fan M-X Wu J-L Li N Guo M-Q. Antioxidant and anti-inflammatory properties of flavonoids from lotus plumule. Food Chem. (2019) 277:706–12. doi: 10.1016/j.foodchem.2018.11.040, PMID: 30502207

[28] Huang YB Lin MW Chao Y Huang CT Tsai YH Wu PC. Anti-oxidant activity and attenuation of bladder hyperactivity by the flavonoid compound kaempferol. Int J Urol. (2013) 21:94–8. doi: 10.1111/iju.12179, PMID: 23634640

[29] Zhang H Li N Li K Li P. Effect of ethanol root extract of *Equisetum arvense* (L) on urinary bladder activity in rats and analysis of principal plant constituents. Trop J Pharm Res. (2015) 14:1451. doi: 10.4314/tjpr.v14i8.18

[30] Chen W-l Zhao J. Association between dietary anthocyanidins intake and depression among US adults: a cross-sectional study (NHANES, 2007–2010 and 2017–2018). BMC Psychiatry. (2023) 23:525. doi: 10.1186/s12888-023-05029-8, PMID: 37474898 PMC10360350

[31] Miyazaki N Katsura R Hamada K Suzutani T. Blueberry prevents the bladder dysfunction in bladder outlet obstruction rats by attenuating oxidative stress and suppressing bladder remodeling. Nutrients. (2020) 12:12. doi: 10.3390/nu12051285, PMID: 32369959 PMC7282255

[32] Schmitt E Stopper H. Estrogenic activity of naturally occurring Anthocyanidins. Nutr Cancer. (2001) 41:145–9. doi: 10.1080/01635581.2001.9680625, PMID: 12094617

[33] Bismantara Aditya Putra Kadek Budi Santosa I Wayan Niryana Nyoman Golden Gede Wirya Kusuma Duarsa Ida Bagus Made Suryawisesa. The effects of purple sweet potato (*Ipomoea Batatas L.*) ethanol extract on bladder urothelial layer and smooth muscle thicknesses in menopausal female Wistar rats. Folia Medica Indones. (2023) 59:180–6. doi: 10.20473/fmi.v59i2.44621

[34] Belwal T Nabavi S Nabavi S Habtemariam S. Dietary Anthocyanins and insulin resistance: when food becomes a medicine. Nutrients. (2017) 9:1111. doi: 10.3390/nu9101111, PMID: 29023424 PMC5691727

[35] Uzun H Yilmaz A Kemik A Zorba OU Kalkan M. Association of Insulin Resistance with overactive bladder in female patients. Int Neurourol J. (2012) 16:181–6. doi: 10.5213/inj.2012.16.4.181, PMID: 23346484 PMC3547179

[36] Leiria LO Sollon C Báu FR Mónica FZ D’Ancona CL De Nucci G. Insulin relaxes bladder via PI3K/AKT/eNOS pathway activation in mucosa: unfolded protein response-dependent insulin resistance as a cause of obesity-associated overactive bladder. J Physiol. (2013) 591:2259–73. doi: 10.1113/jphysiol.2013.251843, PMID: 23478138 PMC3650693

[37] Bendokas V Stanys V Mažeikienė I Trumbeckaite S Baniene R Liobikas J. Anthocyanins: from the field to the antioxidants in the body. Antioxidants. (2020) 9:9. doi: 10.3390/antiox9090819, PMID: 32887513 PMC7555562

[38] Hostetler GL Ralston RA Schwartz SJ. Flavones: food sources, bioavailability, metabolism, and bioactivity. Adv Nutr. (2017) 8:423–35. doi: 10.3945/an.116.012948, PMID: 28507008 PMC5421117

[39] Sweeney P Mutambirwa S Van An N Sharma JB Vanamail P. Flavoxate in the symptomatic treatment of overactive bladder: a meta-analysis. Eur Rev Med Pharmacol Sci. (2016) 20:3073–712. 27649675

[40] Tomoda T Aishima M Takano N Nakano T Seki N Yonemitsu Y. The effects of flavoxate hydrochloride on voltage-dependent L-type Ca2+ currents in human urinary bladder. Br J Pharmacol. (2005) 146:25–32. doi: 10.1038/sj.bjp.0706284, PMID: 15965499 PMC1576239

[41] Mukerji G Yiangou Y Corcoran SL Selmer IS Smith GD Benham CD. Cool and menthol receptor TRPM8 in human urinary bladder disorders and clinical correlations. BMC Urol. (2006) 6:6. doi: 10.1186/1471-2490-6-6, PMID: 16519806 PMC1420318

[42] Sanechika S Shimobori C Ohbuchi K. Identification of herbal components as TRPA1 agonists and TRPM8 antagonists. J Nat Med. (2021) 75:717–25. doi: 10.1007/s11418-021-01515-z, PMID: 33877504

[43] Kostyev FI Vernygorodskyi SV Iatsyna OI. Morphological analysis of interstitial Cajal cells and mast cells in experimental hyperactivity bladder and stress incontinence under influence of pharmacocorrection. Rep Morphol. (2018) 24:5–13. doi: 10.31393/morphology-journal-2018-24(2)-01

