# Supplementary material for: Intake of dietary flavonoids in relation to overactive bladder among U.S. adults: a nutritional strategy for improving urinary health
Source: Front Nutr. 2024 Jul 24;11:1437923. doi: 10.3389/fnut.2024.1437923 (PMC11303291; doi:10.3389/fnut.2024.1437923)
Supplement: Supplementary file 1 [file Data_Sheet_1.docx]

Supplementary Material

# Supplementary Figures and Tables

## Supplementary Tables

**Supplementary Table 1.** Characteristics of participants based on common diseases and other measures by the Overactive Bladder (OAB) status, weighted.

| Characteristics | Overall (*n* = 13,063) | Non-OAB (*n* = 10,223) | OAB (*n* = 2,840) | *P*-value |
| --- | --- | --- | --- | --- |
| **Diseases, *n* (%)** | | | | |
| **DM** |  |  |  | **< 0.0001** |
| No | 9299(77.33) | 7682(81.27) | 1617(63.16) |  |
| DM | 2556(14.09) | 1614(11.50) | 942(28.00) |  |
| IFG | 660(4.74) | 512(4.61) | 148(5.73) |  |
| IGT | 410(2.67) | 315(2.62) | 95(3.11) |  |
| **Hypertension** |  |  |  | **< 0.0001** |
| No | 7273(62.54) | 6262(66.74) | 1011(41.71) |  |
| Yes | 5789(37.46) | 3960(33.26) | 1829(58.29) |  |
| **Hyperlipidemia** |  |  |  | **< 0.0001** |
| No | 3678(30.47) | 3094(32.18) | 584(21.98) |  |
| Yes | 9383(69.53) | 7128(67.82) | 2255(78.02) |  |
| **Cardiovascular Disease** |  |  |  | **< 0.0001** |
| No | 11499(91.15) | 9301(93.42) | 2198(79.97) |  |
| Yes | 1561(8.84) | 921(6.58) | 640(20.03) |  |
| **Stroke** |  |  |  | **< 0.0001** |
| No | 12461(96.60) | 9888(97.70) | 2573(91.90) |  |
| Yes | 581(3.27) | 324(2.30) | 257(8.10) |  |
| **Parkinson** |  |  |  | **< 0.0001** |
| No | 12913(99.05) | 10139(99.32) | 2774(98.01) |  |
| Yes | 142(0.90) | 78(0.68) | 64(1.99) |  |
| **Disease measures, mean (SD)** | | | | |
| PA total time, minutes | 1281.91(34.79) | 1312.45(39.35) | 1095.73(54.13) | **0.002** |
| Sitting time, hours | 5.67(0.06) | 5.66(0.07) | 5.77(0.10) | 0.33 |
| eGFR, mL/min/1.73m^2^ | 91.83(0.49) | 93.23(0.51) | 84.91(0.83) | **< 0.0001** |
| FPG, mmol/L | 5.96(0.04) | 5.87(0.04) | 6.41(0.09) | **< 0.0001** |
| Urine creatinine, mg/dL | 122.00(1.28) | 124.10(1.29) | 111.59(2.60) | **< 0.0001** |
| Uric acid, mg/dL | 5.44(0.03) | 5.43(0.03) | 5.53(0.05) | 0.05 |
| Hs-CRP, mg/L | 3.98(0.20) | 3.70(0.22) | 5.23(0.75) | 0.08 |
| Calcium, mg/dL | 9.38(0.01) | 9.39(0.01) | 9.35(0.02) | **0.01** |
| Blood urea nitrogen, mg/dL | 13.79(0.10) | 13.53(0.10) | 15.06(0.19) | **< 0.0001** |
| **Dietary measures, median (IQR)** | | | | |
| Total energy intake, kcal/day | 1964(1464,2613) | 1987(1479,2654) | 1860(1380,2446) | **< 0.0001** |
| HEI-2015 total score | 50.05(40.55,59.90) | 50.16(40.67,59.97) | 49.64(39.86,59.72) | 0.35 |
| OBS | 20.00(15.00,26.00) | 21.00(15.00,26.00) | 20.00(13.00,25.00) | **< 0.0001** |
| CDAI | -0.35(-2.34, 2.08) | -0.35(-2.29,2.06) | -0.41(-2.57,2.16) | 0.38 |
| DII | 1.69(0.11,2.99) | 1.66(0.07,2.96) | 1.88(0.32,3.11) | **0.002** |

SD, Standard deviation; IQR, Interquartile range; DM, Diabetes Mellitus; IFG, Impaired Fasting Glucose; IGT, Impaired Glucose Tolerance; PA, Physical activity; eGFR, estimated glomerular filtration rate; FPG, Fasting plasma glucose; Hs-CRP: Hypersensitive C-reactive protein; HEI, Healthy eating index; OBS, Oxidative balance score; CDAI, Composite Dietary Antioxidant Index; DII, Dietary Inflammatory Index.

**Supplementary Table 2.** Baseline characteristics of the participants based on the quartiles of the total flavonoid intake, weighted.

|  | Total flavonoid (Range, mg/day) | | | |  |
| --- | --- | --- | --- | --- | --- |
| Variables | Quartile 1  [0,24.625] | Quartile 2  (24.625,64.82] | Quartile 3  (64.82,220.305] | Quartile 4  (220.305,6974.47] | *P*-value |
| **OAB, *n* (%)** |  |  |  |  | 0.52 |
| No | 2500(82.04) | 2553(83.45) | 2573(83.48) | 2597(83.77) |  |
| Yes | 767(17.96) | 712(16.55) | 692(16.52) | 669(16.23) |  |
| **Demographic** | | | | | |
| **Age, *n* (%)** |  |  |  |  | **< 0.0001** |
| 20-41 | 1279(47.74) | 1140(40.95) | 1039(36.39) | 992(35.43) |  |
| 42-61 | 1048(32.68) | 1105(36.67) | 1143(38.19) | 1213(41.80) |  |
| 62-80 | 940(19.58) | 1020(22.38) | 1083(25.42) | 1061(22.77) |  |
| **Sex, *n* (%)** |  |  |  |  | 0.29 |
| Male | 1580(48.02) | 1593(49.09) | 1576(48.50) | 1557(45.89) |  |
| Female | 1687(51.98) | 1672(50.91) | 1689(51.50) | 1709(54.11) |  |
| **Race, *n* (%)** |  |  |  |  | **< 0.0001** |
| Non-Hispanic White | 1485(66.34) | 1406(64.79) | 1390(64.96) | 1727(73.30) |  |
| Mexican American | 513(9.28) | 622(10.89) | 564(9.34) | 341(4.79) |  |
| Non-Hispanic Black | 776(13.68) | 655(11.92) | 645(10.88) | 609(9.06) |  |
| Non-Hispanic Asian | 49(0.95) | 101(1.82) | 136(2.57) | 194(2.78) |  |
| Other/Multi-Racial | 444(9.75) | 481(10.59) | 530(12.25) | 395(10.07) |  |
| **Education level, *n* (%)** |  |  |  |  | **< 0.0001** |
| Less than high school | 422(6.64) | 367(5.57) | 336(4.66) | 190(2.81) |  |
| High school | 1531(46.38) | 1266(35.44) | 1096(30.12) | 1106(31.49) |  |
| College and high | 1310(46.98) | 1631(58.99) | 1827(65.22) | 1967(65.69) |  |
| **Marital status, *n* (%)** |  |  |  |  | **< 0.0001** |
| Not married | 1318(39.05) | 1187(34.72) | 1112(31.24) | 1099(33.24) |  |
| Married | 1948(60.95) | 2076(65.28) | 2153(68.76) | 2163(66.76) |  |
| **Annual household income, *n* (%)** |  |  |  |  | **< 0.0001** |
| < $20,000 | 788(17.95) | 622(13.42) | 590(12.59) | 529(11.82) |  |
| ≥ $20,000 | 2330(82.05) | 2461(86.58) | 2492(87.41) | 2606(88.18) |  |
| **Poverty to income ratio, *n* (%)** |  |  |  |  | **< 0.0001** |
| < 1 | 758(18.61) | 570(13.47) | 524(11.80) | 413(10.07) |  |
| 1-3 | 1428(40.46) | 1339(35.96) | 1217(32.76) | 1178(32.23) |  |
| ≥ 3 | 807(40.93) | 1034(50.56) | 1196(55.44) | 1398(57.70) |  |
| **Weight status, *n* (%)** |  |  |  |  | **< 0.0001** |
| Normal (BMI＜25) | 790(25.62) | 863(27.46) | 888(29.45) | 908(31.18) |  |
| Overweight (25≤BMI＜30) | 983(28.76) | 1092(33.18) | 1156(34.09) | 1099(33.15) |  |
| Obese (BMI≥30) | 1457(45.62) | 1285(39.36) | 1198(36.46) | 1236(35.68) |  |
| **Alcohol status, *n* (%)** |  |  |  |  | **0.01** |
| Never | 386(10.12) | 393(9.64) | 439(11.14) | 418(10.44) |  |
| Former | 558(14.25) | 424(10.56) | 385(9.29) | 457(11.15) |  |
| Current | 2050(75.63) | 2247(79.80) | 2241(79.58) | 2213(78.41) |  |
| **Smoking status, *n* (%)** |  |  |  |  | **< 0.0001** |
| Never | 1495(45.40) | 1812(58.27) | 1947(61.48) | 1851(58.00) |  |
| Former | 797(24.29) | 834(24.49) | 859(25.12) | 844(25.68) |  |
| Current | 975(30.32) | 619(17.24) | 458(13.40) | 571(16.32) |  |
| **Caffeine intake, median (IQR)** | 108.50(32.00,223.00) | 98.50(22.50,213.00) | 102.50(22.50,218.50) | 161.50(84.50,283.50) | **< 0.0001** |
| **Disease measures, mean (SD)** | | | | | |
| PA total time, minutes | 1514.04(63.91) | 1255.96(45.10) | 1171.67(44.42) | 1220.79(48.07) | **< 0.0001** |
| Sitting time, hours | 5.63(0.10) | 5.67(0.09) | 5.67(0.12) | 5.72(0.09) | 0.88 |
| eGFR, mL/min/1.73m^2^ | 93.14(0.80) | 92.25(0.81) | 91.64(0.79) | 90.56(0.64) | **0.04** |
| FPG, mmol/L | 5.96(0.05) | 6.03(0.07) | 5.95(0.08) | 5.92(0.06) | 0.65 |
| Urine creatinine, mg/dL | 134.20(2.76) | 119.37(2.49) | 117.63(2.15) | 117.82(2.57) | **< 0.0001** |
| Uric acid, mg/dL | 5.52(0.05) | 5.42(0.04) | 5.42(0.05) | 5.42(0.04) | 0.39 |
| Hs-CRP, mg/L | 5.23(0.78) | 3.45(0.30) | 3.45(0.13) | 3.77(0.34) | 0.22 |
| Calcium, mg/dL | 9.37(0.02) | 9.39(0.02) | 9.40(0.02) | 9.37(0.02) | 0.15 |
| Blood urea nitrogen, mg/dL | 13.27(0.18) | 13.90(0.13) | 14.29(0.15) | 13.68(0.13) | **< 0.0001** |
| **Dietary measures, median (IQR)** | | | | | |
| Total energy intake, kcal/day | 1765(1284,2351) | 1961 (1442,2642) | 2123 (1615,2736) | 2015 (1547,2701) | **< 0.0001** |
| HEI-2015 total score | 42.24(34.62,50.35) | 51.12(42.55,59.50) | 55.02(45.91,64.41) | 52.29(41.97,62.22) | **< 0.0001** |
| OBS | 16.00(11.00,22.00) | 20.00(15.00,26.00) | 23.00(17.00,28.00) | 22.00(16.00,27.00) | **< 0.0001** |
| CDAI | -1.64(-3.46,0.34) | -0.36(-2.26,1.79) | 0.4(-1.43,2.96) | 0.11(-1.98,2.76) | **< 0.0001** |
| DII | 2.70(1.51,3.63) | 1.67(0.26,2.81) | 0.92(-0.57,2.30) | 1.34(-0.29,2.81) | **< 0.0001** |

SD, Standard deviation; IQR, Interquartile range; BMI, Body mass index; DM, Diabetes Mellitus; IFG, Impaired Fasting Glucose; IGT, Impaired Glucose Tolerance; PA, Physical activity; eGFR, estimated glomerular filtration rate; FPG, Fasting plasma glucose; Hs-CRP: Hypersensitive C-reactive protein; HEI, Healthy eating index; OBS, Oxidative balance score; CDAI, Composite Dietary Antioxidant Index; DII, Dietary Inflammatory Index.

**Supplementary Table 3.** Baseline characteristics of the participants based on the quartiles of the anthocyanidin intake, weighted.

|  | Anthocyanidin (Range, mg/day) | | | |  |
| --- | --- | --- | --- | --- | --- |
| Variables | Quartile 1  [0,0.12] | Quartile 2  (0.12,2.06] | Quartile 3  (2.06,11.07] | Quartile 4  (11.07,756.1] | *P*-value |
| **OAB, *n* (%)** |  |  |  |  | **0.02** |
| No | 2516(81.17) | 2552(82.80) | 2508(82.97) | 2647(85.61) |  |
| Yes | 748(18.83) | 717(17.20) | 757(17.03) | 618(14.39) |  |
| **Demographic** | | | | | |
| **Age, *n* (%)** |  |  |  |  | **< 0.0001** |
| 20-41 | 1330(48.00) | 1137(41.83) | 1062(38.79) | 921(31.91) |  |
| 42-61 | 1098(34.62) | 1114(37.52) | 1121(37.11) | 1176(40.59) |  |
| 62-80 | 836(17.38) | 1018(20.64) | 1082(24.10) | 1168(27.51) |  |
| **Sex, *n* (%)** |  |  |  |  | **< 0.0001** |
| Male | 1770(53.74) | 1607(48.80) | 1526(48.80) | 1403(40.72) |  |
| Female | 1494(46.26) | 1662(51.20) | 1739(51.20) | 1862(59.28) |  |
| **Race, *n* (%)** |  |  |  |  | **< 0.0001** |
| Non-Hispanic White | 1564(67.47) | 1325(62.90) | 1358(63.76) | 1761(74.86) |  |
| Mexican American | 348(6.72) | 686(11.82) | 645(10.37) | 361(5.45) |  |
| Non-Hispanic Black | 862(14.66) | 680(12.05) | 622(11.37) | 521(7.49) |  |
| Non-Hispanic Asian | 92(1.54) | 108(2.19) | 103(1.86) | 177(2.63) |  |
| Other/Multi-Racial | 398(9.60) | 470(11.04) | 537(12.64) | 445(9.57) |  |
| **Education level, *n* (%)** |  |  |  |  | **< 0.0001** |
| Less than high school | 275(4.46) | 425(6.31) | 422(5.92) | 193(2.99) |  |
| High school | 1550(44.62) | 1348(40.93) | 1174(34.86) | 927(23.82) |  |
| College and high | 1437(50.91) | 1489(52.76) | 1666(59.22) | 2143(73.19) |  |
| **Marital status, *n* (%)** |  |  |  |  | **< 0.0001** |
| Not married | 1358(40.56) | 1185(34.82) | 1096(31.92) | 1077(30.90) |  |
| Married | 1906(59.44) | 2080(65.18) | 2168(68.08) | 2186(69.10) |  |
| **Annual household income, *n* (%)** |  |  |  |  | **< 0.0001** |
| < $20,000 | 779(17.75) | 685(15.31) | 611(13.47) | 454(9.43) |  |
| ≥ $20,000 | 2334(82.25) | 2404(84.69) | 2488(86.53) | 2663(90.57) |  |
| **Poverty to income ratio, *n* (%)** |  |  |  |  | **< 0.0001** |
| < 1 | 740(18.42) | 625(15.51) | 546(12.72) | 354(7.48) |  |
| 1-3 | 1362(38.63) | 1383(38.86) | 1331(36.40) | 1086(28.14) |  |
| ≥ 3 | 897(42.95) | 933(45.63) | 1060(50.88) | 1545(64.38) |  |
| **Weight status, *n* (%)** |  |  |  |  | **< 0.0001** |
| Normal (BMI＜25) | 829(25.73) | 797(26.05) | 827(27.94) | 996(33.69) |  |
| Overweight (25≤BMI＜30) | 947(28.06) | 1104(31.73) | 1115(33.82) | 1164(35.44) |  |
| Obese (BMI≥30) | 1455(46.22) | 1343(42.22) | 1293(38.24) | 1085(30.87) |  |
| **Alcohol status, *n* (%)** |  |  |  |  | **< 0.0001** |
| Never | 364(8.91) | 443(10.67) | 462(12.89) | 367(9.23) |  |
| Former | 483(12.39) | 509(13.49) | 499(11.48) | 333(8.36) |  |
| Current | 2144(78.70) | 2108(75.83) | 2100(75.63) | 2399(82.41) |  |
| **Smoking status, *n* (%)** |  |  |  |  | **< 0.0001** |
| Never | 1494(46.81) | 1726(54.10) | 1913(59.24) | 1972(62.77) |  |
| Former | 732(22.27) | 826(24.30) | 853(25.63) | 923(27.26) |  |
| Current | 1038(30.92) | 717(21.60) | 498(15.14) | 370(9.97) |  |
| **Caffeine intake, median (IQR)** | 123.00(46.50,243.00) | 117.00(41.50,228.00) | 109.50(37.50,222.50) | 138.50(43.50,250.00) | **0.002** |
| **Disease measures, mean (SD)** | | | | | |
| PA total time, minutes | 1567.38(76.59) | 1328.86(62.22) | 1228.46(56.22) | 1049.95(37.44) | **< 0.0001** |
| Sitting time, hours | 5.64(0.08) | 5.46(0.10) | 5.66(0.10) | 5.90(0.10) | **0.01** |
| eGFR, mL/min/1.73m^2^ | 92.67(0.67) | 93.17(0.73) | 91.83(0.87) | 89.99(0.63) | **0.002** |
| FPG, mmol/L | 5.90(0.04) | 6.01(0.05) | 5.96(0.05) | 5.98(0.08) | 0.29 |
| Urine creatinine, mg/dL | 135.04(2.22) | 124.38(1.79) | 123.65(2.33) | 106.97(2.23) | **< 0.0001** |
| Uric acid, mg/dL | 5.63(0.04) | 5.47(0.04) | 5.45(0.04) | 5.25(0.04) | **< 0.0001** |
| Hs-CRP, mg/L | 4.56(0.46) | 4.61(0.62) | 3.75(0.26) | 3.13(0.25) | **0.004** |
| Calcium, mg/dL | 9.37(0.02) | 9.37(0.02) | 9.39(0.01) | 9.40(0.02) | 0.36 |
| Blood urea nitrogen, mg/dL | 13.28(0.14) | 13.59(0.11) | 13.94(0.19) | 14.26(0.15) | **< 0.0001** |
| **Dietary measures, median (IQR)** | | | | | |
| Total energy intake, kcal/day | 1894(1367,2608) | 1966(1477,2579) | 1987(1483,2636) | 1998(1516,2651) | 0.02 |
| HEI-2015 total score | 41.84(34.17,50.32) | 48.45(39.90,57.26) | 52.18(43.26,61.81) | 57.56(48.56,66.52) | **< 0.0001** |
| OBS | 17.00(12.00,23.00) | 20.00(14.00,25.00) | 21.00(16.00,26.00) | 24.00(18.00,28.00) | **< 0.0001** |
| CDAI | -1.40(-3.23,0.75) | -0.45(-2.48,1.66) | -0.29(-2.07,2.02) | 0.64(-1.31,3.34) | **< 0.0001** |
| DII | 2.55(1.19,3.54) | 1.87(0.47,3.06) | 1.49(0.09,2.83) | 0.80(-0.72,2.29) | **< 0.0001** |

SD, Standard deviation; IQR, Interquartile range; BMI, Body mass index; DM, Diabetes Mellitus; IFG, Impaired Fasting Glucose; IGT, Impaired Glucose Tolerance; PA, Physical activity; eGFR, estimated glomerular filtration rate; FPG, Fasting plasma glucose; Hs-CRP: Hypersensitive C-reactive protein; HEI, Healthy eating index; OBS, Oxidative balance score; CDAI, Composite Dietary Antioxidant Index; DII, Dietary Inflammatory Index.

**Supplementary Table 4.** Baseline characteristics of the participants based on the quartiles of the flavone intake, weighted.

|  | Flavone (Range, mg/day) | | | |  |
| --- | --- | --- | --- | --- | --- |
| Variables | Quartile 1  [0,0.185] | Quartile 2  (0.185,0.51] | Quartile 3  (0.51,1.09] | Quartile 4  (1.09,87.245] | *P*-value |
| **OAB, *n* (%)** |  |  |  |  | **< 0.001** |
| No | 2512(80.04) | 2514(83.43) | 2573(83.81) | 2624(85.37) |  |
| Yes | 813(19.96) | 715(16.57) | 685(16.19) | 627(14.63) |  |
| **Demographic** | | | | | |
| **Age, *n* (%)** |  |  |  |  | **0.01** |
| 20-41 | 1245(43.81) | 1118(41.64) | 1073(38.78) | 1014(35.78) |  |
| 42-61 | 1073(35.23) | 1071(35.41) | 1130(37.68) | 1235(41.41) |  |
| 62-80 | 1007(20.97) | 1040(22.95) | 1055(23.54) | 1002(22.81) |  |
| **Sex, *n* (%)** |  |  |  |  | **0.004** |
| Male | 1686(49.78) | 1474(43.88) | 1530(46.43) | 1616(50.62) |  |
| Female | 1639(50.22) | 1755(56.12) | 1728(53.57) | 1635(49.38) |  |
| **Race, *n* (%)** |  |  |  |  | **< 0.0001** |
| Non-Hispanic White | 1508(65.51) | 1481(65.82) | 1479(68.85) | 1540(69.94) |  |
| Mexican American | 373(7.09) | 493(8.47) | 583(9.20) | 591(8.79) |  |
| Non-Hispanic Black | 899(15.55) | 725(13.57) | 590(9.92) | 471(6.63) |  |
| Non-Hispanic Asian | 56(1.12) | 82(1.48) | 126(2.15) | 216(3.37) |  |
| Other/Multi-Racial | 489(10.73) | 448(10.67) | 480(9.88) | 433(11.27) |  |
| **Education level, *n* (%)** |  |  |  |  | **< 0.0001** |
| Less than high school | 366(5.87) | 378(5.76) | 297(4.16) | 274(3.66) |  |
| High school | 1576(44.88) | 1309(40.31) | 1154(33.22) | 960(25.31) |  |
| College and high | 1379(49.25) | 1537(53.93) | 1805(62.62) | 2014(71.03) |  |
| **Marital status, *n* (%)** |  |  |  |  | **< 0.0001** |
| Not married | 1388(38.91) | 1222(36.91) | 1111(33.52) | 995(29.21) |  |
| Married | 1935(61.09) | 2007(63.09) | 2142(66.48) | 2256(70.79) |  |
| **Annual household income, *n* (%)** |  |  |  |  | **< 0.0001** |
| ＜$20,000 | 823(18.76) | 686(16.86) | 567(11.49) | 453(8.99) |  |
| ≥$20,000 | 2322(81.24) | 2387(83.14) | 2540(88.51) | 2640(91.01) |  |
| **Poverty to income ratio, *n* (%)** |  |  |  |  | **< 0.0001** |
| < 1 | 792(19.38) | 584(14.92) | 480(10.42) | 409(9.25) |  |
| 1-3 | 1439(40.40) | 1353(39.33) | 1232(32.85) | 1138(29.18) |  |
| ≥3 | 796(40.22) | 979(45.74) | 1259(56.73) | 1401(61.57) |  |
| **Weight status, *n* (%)** |  |  |  |  | **0.002** |
| Normal (BMI＜25) | 906(28.27) | 804(26.95) | 837(27.36) | 902(31.35) |  |
| Overweight (25≤BMI＜30) | 976(28.69) | 1120(32.76) | 1122(34.13) | 1112(33.65) |  |
| Obese (BMI≥30) | 1407(43.04) | 1275(40.28) | 1275(38.51) | 1219(35.00) |  |
| **Alcohol status, *n* (%)** |  |  |  |  | **0.004** |
| Never | 429(11.97) | 451(11.34) | 396(9.38) | 360(9.00) |  |
| Former | 504(12.24) | 513(12.79) | 437(11.30) | 370(9.08) |  |
| Current | 2080(75.79) | 2073(75.87) | 2269(79.32) | 2329(81.92) |  |
| **Smoking status, *n* (%)** |  |  |  |  | **< 0.0001** |
| Never | 1599(49.12) | 1712(54.37) | 1872(59.76) | 1922(59.93) |  |
| Former | 752(22.39) | 829(25.45) | 861(24.78) | 892(26.94) |  |
| Current | 974(28.49) | 688(20.18) | 524(15.46) | 437(13.13) |  |
| **Caffeine intake, median (IQR)** | 103.50(31.50,223.50) | 113.00(41.00,224.00) | 126.50(44.50,234.00) | 144.00(55.00,263.00) | **< 0.001** |
| **Disease measures, mean (SD)** | | | | | |
| PA total time, minutes | 1430.81(54.80) | 1276.88(60.21) | 1255.00(64.26) | 1188.51(46.41) | **0.004** |
| Sitting time, hours | 5.52(0.11) | 5.71(0.07) | 5.65(0.09) | 5.81(0.10) | 0.1 |
| eGFR, mL/min/1.73m^2^ | 92.41(0.56) | 91.58(0.88) | 91.82(0.81) | 91.53(0.69) | 0.54 |
| FPG, mmol/L | 6.00(0.08) | 5.97(0.06) | 5.91(0.06) | 5.96(0.05) | 0.66 |
| Urine creatinine, mg/dL | 137.99(2.39) | 126.20(2.67) | 115.50(2.43) | 109.95(1.88) | **< 0.0001** |
| Uric acid, mg/dL | 5.46(0.04) | 5.41(0.05) | 5.46(0.04) | 5.44(0.04) | 0.86 |
| Hs-CRP, mg/L | 4.65(0.18) | 4.72(0.79) | 3.63(0.42) | 2.93(0.16) | **< 0.0001** |
| Calcium, mg/dL | 9.36(0.02) | 9.38(0.02) | 9.39(0.01) | 9.40(0.02) | 0.09 |
| Blood urea nitrogen, mg/dL | 13.30(0.15) | 13.76(0.17) | 13.73(0.13) | 14.30(0.16) | **< 0.0001** |
| **Dietary measures, median (IQR)** | | | | | |
| Total energy intake, kcal/day | 1810(1306,2417) | 1897 (1423,2548) | 1978 (1493,2668) | 2150 (1675,2793) | **< 0.0001** |
| HEI-2015 total score | 43.72(35.92,52.42) | 47.98(39.16,57.00) | 51.81(41.98,61.09) | 56.28(47.23,66.28) | **< 0.0001** |
| OBS | 16.00(11.00,22.00) | 19.00(14.00,24.00) | 21.00(16.00,26.00) | 25.00(19.00,29.00) | **< 0.0001** |
| CDAI | -1.74(-3.46,0.28) | -0.86(-2.63,1.27) | -0.15(-1.85,2.26) | 1.24(-0.84,4.12) | **< 0.0001** |
| DII | 2.70(1.49,3.60) | 2.10(0.69,3.21) | 1.39(-0.02,2.75) | 0.36(-1.01,1.93) | **< 0.0001** |

SD, Standard deviation; IQR, Interquartile range; BMI, Body mass index; DM, Diabetes Mellitus; IFG, Impaired Fasting Glucose; IGT, Impaired Glucose Tolerance; PA, Physical activity; eGFR, estimated glomerular filtration rate; FPG, Fasting plasma glucose; Hs-CRP: Hypersensitive C-reactive protein; HEI, Healthy eating index; OBS, Oxidative balance score; CDAI, Composite Dietary Antioxidant Index; DII, Dietary Inflammatory Index.

**Supplementary Table 5.** Baseline characteristics of the participants based on the quartiles of the flavonol intake, weighted.

|  | Flavonol (Range, mg/day) | | | |  |
| --- | --- | --- | --- | --- | --- |
| Variables | Quartile 1  [0,6.932] | Quartile 2  (6.932,12.745] | Quartile 3  (12.745,22.398] | Quartile 4  (22.398,332.035] | *P*-value |
| **OAB, *n* (%)** |  |  |  |  | **0.004** |
| No | 2444(80.56) | 2545(83.11) | 2588(83.48) | 2646(85.18) |  |
| Yes | 822(19.44) | 719(16.89) | 679(16.52) | 620(14.82) |  |
| **Demographic** | | | | | |
| **Age, *n* (%)** |  |  |  |  | **< 0.0001** |
| 20-41 | 1158(43.15) | 1121(41.25) | 1146(41.01) | 1025(35.11) |  |
| 42-61 | 1000(33.24) | 1101(35.31) | 1125(36.10) | 1283(44.16) |  |
| 62-80 | 1108(23.61) | 1042(23.45) | 996(22.89) | 958(20.73) |  |
| **Sex, *n* (%)** |  |  |  |  | **< 0.0001** |
| Male | 1355(40.52) | 1522(46.65) | 1597(47.78) | 1832(54.54) |  |
| Female | 1911(59.48) | 1742(53.35) | 1670(52.22) | 1434(45.46) |  |
| **Race, *n* (%)** |  |  |  |  | **< 0.0001** |
| Non-Hispanic White | 1410(65.13) | 1432(65.01) | 1470(66.67) | 1696(72.60) |  |
| Mexican American | 492(9.28) | 544(9.22) | 584(9.19) | 420(6.30) |  |
| Non-Hispanic Black | 803(14.89) | 721(12.73) | 625(10.52) | 536(7.85) |  |
| Non-Hispanic Asian | 58(1.08) | 93(1.85) | 132(2.29) | 197(2.84) |  |
| Other/Multi-Racial | 503(9.62) | 474(11.19) | 456(11.33) | 417(10.41) |  |
| **Education level, *n* (%)** |  |  |  |  | **< 0.0001** |
| Less than high school | 420(6.56) | 375(5.76) | 319(4.74) | 201(2.73) |  |
| High school | 1453(43.48) | 1286(36.84) | 1154(33.01) | 1106(30.66) |  |
| College and high | 1390(49.96) | 1600(57.39) | 1789(62.25) | 1956(66.61) |  |
| **Marital status, *n* (%)** |  |  |  |  | **< 0.0001** |
| Not married | 1353(40.22) | 1214(34.56) | 1112(32.61) | 1037(31.51) |  |
| Married | 1912(59.78) | 2049(65.44) | 2154(67.39) | 2225(68.49) |  |
| **Annual household income, *n* (%)** |  |  |  |  | **< 0.0001** |
| ＜$20,000 | 763(18.15) | 670(15.04) | 598(12.76) | 498(10.43) |  |
| ≥$20,000 | 2325(81.85) | 2419(84.96) | 2520(87.24) | 2625(89.57) |  |
| **Poverty to income ratio, *n* (%)** |  |  |  |  | **< 0.0001** |
| < 1 | 695(17.60) | 625(15.55) | 520(11.91) | 425(9.47) |  |
| 1-3 | 1432(40.33) | 1301(36.60) | 1284(33.91) | 1145(31.22) |  |
| ≥3 | 824(42.08) | 1026(47.85) | 1167(54.18) | 1418(59.31) |  |
| **Weight status, *n* (%)** |  |  |  |  | **< 0.001** |
| Normal (BMI＜25) | 824(27.52) | 819(26.97) | 878(28.04) | 928(31.20) |  |
| Overweight (25≤BMI＜30) | 984(28.83) | 1094(32.07) | 1139(33.82) | 1113(34.03) |  |
| Obese (BMI≥30) | 1420(43.65) | 1316(40.97) | 1232(38.14) | 1208(34.76) |  |
| **Alcohol status, *n* (%)** |  |  |  |  | **< 0.0001** |
| Never | 478(12.93) | 427(11.54) | 395(9.53) | 336(8.15) |  |
| Former | 576(14.45) | 465(12.49) | 396(9.72) | 387(9.24) |  |
| Current | 1929(72.62) | 2145(75.97) | 2296(80.75) | 2381(82.61) |  |
| **Smoking status, *n* (%)** |  |  |  |  | **< 0.001** |
| Never | 1677(51.89) | 1818(57.80) | 1839(57.00) | 1771(56.62) |  |
| Former | 819(24.00) | 810(23.82) | 842(26.50) | 863(25.18) |  |
| Current | 770(24.11) | 635(18.38) | 586(16.50) | 632(18.20) |  |
| **Caffeine intake, mg/day, median (IQR)** | 84.50(16.00,195.50) | 99.50(26.00,208.00) | 117.00(42.50,222.00) | 172.50(87.00,297.00) | **< 0.0001** |
| **Disease measures, mean (SD)** | | | | | |
| PA total time, minutes | 1350.15(69.68) | 1231.90(43.08) | 1201.05(57.90) | 1345.62(62.04) | 0.16 |
| Sitting time, hours | 5.69(0.09) | 5.59(0.11) | 5.74(0.08) | 5.67(0.11) | 0.67 |
| eGFR, mL/min/1.73m^2^ | 91.97(0.82) | 91.67(0.81) | 91.89(0.54) | 91.79(0.67) | 0.99 |
| FPG, mmol/L | 5.97(0.07) | 6.05(0.08) | 5.86(0.05) | 5.98(0.05) | 0.07 |
| Urine creatinine, mg/dL | 131.25(2.34) | 126.33(2.48) | 117.80(2.01) | 114.93(2.48) | **< 0.0001** |
| Uric acid, mg/dL | 5.34(0.04) | 5.41(0.05) | 5.44(0.04) | 5.55(0.05) | **0.02** |
| Hs-CRP, mg/L | 4.71(0.64) | 3.91(0.29) | 4.04(0.55) | 3.33(0.25) | 0.16 |
| Calcium, mg/dL | 9.37(0.02) | 9.40(0.01) | 9.38(0.02) | 9.38(0.02) | 0.31 |
| Blood urea nitrogen, mg/dL | 13.59(0.17) | 13.71(0.19) | 14.02(0.12) | 13.79(0.15) | 0.14 |
| **Dietary measures, median (IQR)** | | | | | |
| Total energy intake, kcal/day | 1611.00(1179.00,2192.00) | 1914.00(1455.00,2487.00) | 2065.00(1585.00,2692.00) | 2249.00(1706.00,2989.00) | **< 0.0001** |
| HEI-2015 total score | 43.92(36.06,52.82) | 49.49(40.12,59.86) | 51.84(42.27,61.48) | 53.83(44.09,63.13) | **< 0.0001** |
| OBS | 15.00(11.00,21.00) | 20.00(14.00,25.00) | 22.00(16.00,26.00) | 24.00(19.00,28.00) | **< 0.0001** |
| CDAI | -1.98(-3.60,0.06) | -0.65(-2.37,1.54) | 0.10(-1.81,2.39) | 0.84(-1.18,3.75) | **< 0.0001** |
| DII | 2.82(1.64,3.70) | 1.90(0.48,2.98) | 1.33(0.00,2.67) | 0.72(-0.79,2.16) | **< 0.0001** |

SD, Standard deviation; IQR, Interquartile range; BMI, Body mass index; DM, Diabetes Mellitus; IFG, Impaired Fasting Glucose; IGT, Impaired Glucose Tolerance; PA, Physical activity; eGFR, estimated glomerular filtration rate; FPG, Fasting plasma glucose; Hs-CRP: Hypersensitive C-reactive protein; HEI, Healthy eating index; OBS, Oxidative balance score; CDAI, Composite Dietary Antioxidant Index; DII, Dietary Inflammatory Index.

**Supplementary Table 6.** Baseline characteristics of the participants based on the quartiles of the isoflavone intake, weighted.

|  | Isoflavone (Range, mg/day) | | | |  |
| --- | --- | --- | --- | --- | --- |
| Variables | Quartile 1  [0,0] | Quartile 2  (0,0.01] | Quartile 3  (0.01,0.075] | Quartile 4  (0.075,390.6] | *P*-value |
| **OAB, *n* (%)** |  |  |  |  | **< 0.001** |
| No | 3768(81.03) | 1366(84.21) | 2422(82.88) | 2667(86.05) |  |
| Yes | 1176(18.97) | 376(15.79) | 665(17.12) | 623(13.95) |  |
| **Demographic** | | | | | |
| **Age, *n* (%)** |  |  |  |  | **< 0.001** |
| 20-41 | 1674(39.34) | 528(35.78) | 996(38.02) | 1252(44.27) |  |
| 42-61 | 1662(37.02) | 596(39.40) | 1040(37.28) | 1211(37.58) |  |
| 62-80 | 1608(23.64) | 618(24.81) | 1051(24.70) | 827(18.15) |  |
| **Sex, *n* (%)** |  |  |  |  | 0.15 |
| Male | 2361(47.11) | 788(46.12) | 1514(47.17) | 1643(50.09) |  |
| Female | 2583(52.89) | 954(53.88) | 1573(52.83) | 1647(49.91) |  |
| **Race, *n* (%)** |  |  |  |  | **< 0.0001** |
| Non-Hispanic White | 2453(69.25) | 835(68.74) | 1344(67.39) | 1376(64.93) |  |
| Mexican American | 604(7.06) | 222(7.62) | 641(10.45) | 573(8.96) |  |
| Non-Hispanic Black | 1179(13.26) | 398(12.15) | 547(9.78) | 561(9.29) |  |
| Non-Hispanic Asian | 109(1.43) | 37(1.05) | 115(2.24) | 219(3.34) |  |
| Other/Multi-Racial | 599(9.01) | 250(10.43) | 440(10.13) | 561(13.49) |  |
| **Education level, *n* (%)** |  |  |  |  | **< 0.0001** |
| Less than high school | 502(5.46) | 147(3.87) | 383(5.90) | 283(3.50) |  |
| High school | 2152(41.89) | 659(35.02) | 1152(33.15) | 1036(29.17) |  |
| College and high | 2288(52.65) | 932(61.11) | 1547(60.96) | 1968(67.33) |  |
| **Marital status, *n* (%)** |  |  |  |  | 0.11 |
| Not married | 1903(36.36) | 638(32.80) | 1037(32.32) | 1138(34.49) |  |
| Married | 3039(63.64) | 1103(67.20) | 2048(67.68) | 2150(65.51) |  |
| **Annual household income, *n* (%)** |  |  |  |  | **< 0.0001** |
| ＜$20,000 | 1093(16.33) | 352(14.43) | 579(12.78) | 505(10.95) |  |
| ≥$20,000 | 3616(83.67) | 1301(85.57) | 2355(87.22) | 2617(89.05) |  |
| **Poverty to income ratio, *n* (%)** |  |  |  |  | **< 0.001** |
| < 1 | 947(15.16) | 292(12.35) | 527(12.96) | 499(11.64) |  |
| 1-3 | 2028(37.52) | 706(35.88) | 1226(35.10) | 1202(31.76) |  |
| ≥3 | 1535(47.32) | 577(51.78) | 1030(51.93) | 1293(56.60) |  |
| **Weight status, *n* (%)** |  |  |  |  | **< 0.0001** |
| Normal (BMI＜25) | 1255(26.59) | 459(28.53) | 752(26.45) | 983(33.11) |  |
| Overweight (25≤BMI＜30) | 1575(31.18) | 560(30.61) | 1095(34.52) | 1100(33.03) |  |
| Obese (BMI≥30) | 2071(42.22) | 701(40.86) | 1213(39.03) | 1191(33.86) |  |
| **Alcohol status, *n* (%)** |  |  |  |  | **0.01** |
| Never | 636(11.70) | 229(8.87) | 388(9.37) | 383(10.02) |  |
| Former | 778(12.87) | 238(10.30) | 439(11.33) | 369(9.45) |  |
| Current | 3212(75.42) | 1156(80.83) | 2072(79.30) | 2311(80.53) |  |
| **Smoking status, *n* (%)** |  |  |  |  | **< 0.0001** |
| Never | 2514(50.95) | 959(56.91) | 1714(57.47) | 1918(61.11) |  |
| Former | 1253(25.27) | 441(23.05) | 821(26.48) | 819(24.12) |  |
| Current | 1177(23.79) | 342(20.04) | 552(16.05) | 552(14.77) |  |
| **Caffeine intake, mg/day, median (IQR)** | 121.00(40.00,235.00) | 124.50(47.50,255.00) | 130.00(50.50,245.50) | 113.50(36.00,222.00) | **0.02** |
| **Disease measures, mean (SD)** | | | | | |
| PA total time, minutes | 1333.63(54.15) | 1142.97(55.32) | 1284.26(52.42) | 1279.27(66.36) | 0.06 |
| Sitting time, hours | 5.53(0.09) | 5.79(0.11) | 5.63(0.11) | 5.86(0.10) | 0.06 |
| eGFR, mL/min/1.73m^2^ | 90.78(0.60) | 90.87(0.81) | 92.30(0.77) | 93.39(0.82) | **0.02** |
| FPG, mmol/L | 5.95(0.05) | 6.21(0.12) | 6.01(0.07) | 5.80(0.04) | **0.01** |
| Urine creatinine, mg/dL | 126.77(1.91) | 121.04(2.85) | 118.59(2.13) | 118.68(1.71) | **0.003** |
| Uric acid, mg/dL | 5.50(0.04) | 5.40(0.05) | 5.45(0.05) | 5.37(0.04) | 0.1 |
| Hs-CRP, mg/L | 4.70(0.44) | 4.17(0.27) | 3.62(0.29) | 3.27(0.25) | **0.01** |
| Calcium, mg/dL | 9.39(0.02) | 9.38(0.02) | 9.39(0.02) | 9.37(0.02) | 0.48 |
| Blood urea nitrogen, mg/dL | 13.53(0.11) | 14.08(0.20) | 13.85(0.20) | 13.94(0.17) | **0.003** |
| **Dietary measures, median (IQR)** | | | | | |
| Total energy intake, kcal/day | 1868.00(1381.00,2509.00) | 1915.00(1403.00,2537.00) | 2008.00(1511.00,2657.00) | 2095.00(1561.00,2799.00) | **< 0.0001** |
| HEI-2015 total score | 46.54(37.62,55.95) | 50.35(41.92,59.54) | 52.30(43.41,61.33) | 52.91(43.04,63.72) | **< 0.0001** |
| OBS | 18.00(13.00,24.00) | 20.00(14.00,25.00) | 22.00(16.00,27.00) | 23.00(17.00,28.00) | **< 0.0001** |
| CDAI | -1.02(-2.87,1.10) | -0.25(-2.39,1.75) | 0.18(-1.83,2.61) | 0.25(-1.76,3.20) | **< 0.0001** |
| DII | 2.26(0.84,3.32) | 1.81(0.34,3.08) | 1.29(-0.09,2.65) | 1.02(-0.58,2.53) | **< 0.0001** |

SD, Standard deviation; IQR, Interquartile range; BMI, Body mass index; DM, Diabetes Mellitus; IFG, Impaired Fasting Glucose; IGT, Impaired Glucose Tolerance; PA, Physical activity; eGFR, estimated glomerular filtration rate; FPG, Fasting plasma glucose; Hs-CRP: Hypersensitive C-reactive protein; HEI, Healthy eating index; OBS, Oxidative balance score; CDAI, Composite Dietary Antioxidant Index; DII, Dietary Inflammatory Index.

**Supplementary Table 7.** Baseline characteristics of the participants based on the quartiles of the flavanone intake, weighted.

|  | Flavanone (Range, mg/day) | | | |  |
| --- | --- | --- | --- | --- | --- |
| Variables | Quartile 1  [0,0.06] | Quartile 2  (0.06,0.62] | Quartile 3  (0.62,18.985] | Quartile 4  (18.985,590.625] | *P*-value |
| **OAB, *n* (%)** |  |  |  |  | 0.08 |
| No | 2507(82.16) | 2562(83.01) | 2617(85.24) | 2537(82.21) |  |
| Yes | 778(17.84) | 691(16.99) | 642(14.76) | 729(17.79) |  |
| **Demographic** | | | | | |
| **Age, *n* (%)** |  |  |  |  | **< 0.001** |
| 20-41 | 1185(44.30) | 1136(40.00) | 1131(39.21) | 998(35.67) |  |
| 42-61 | 1124(36.49) | 1159(38.64) | 1135(37.39) | 1091(37.65) |  |
| 62-80 | 976(19.20) | 958(21.36) | 993(23.39) | 1177(26.68) |  |
| **Sex, *n* (%)** |  |  |  |  | **< 0.0001** |
| Male | 1670(50.45) | 1574(48.88) | 1403(41.27) | 1659(51.29) |  |
| Female | 1615(49.55) | 1679(51.12) | 1856(58.73) | 1607(48.71) |  |
| **Race, *n* (%)** |  |  |  |  | **< 0.0001** |
| Non-Hispanic White | 1595(68.18) | 1569(69.68) | 1510(69.72) | 1334(62.12) |  |
| Mexican American | 377(6.90) | 482(7.79) | 582(8.60) | 599(10.51) |  |
| Non-Hispanic Black | 783(13.31) | 609(9.74) | 593(9.30) | 700(13.10) |  |
| Non-Hispanic Asian | 119(2.05) | 141(2.48) | 95(1.35) | 125(2.46) |  |
| Other/Multi-Racial | 411(9.56) | 452(10.30) | 479(11.02) | 508(11.81) |  |
| **Education level, *n* (%)** |  |  |  |  | **< 0.0001** |
| Less than high school | 382(5.68) | 294(4.43) | 267(3.46) | 372(5.92) |  |
| High school | 1502(45.98) | 1304(35.73) | 1080(29.35) | 1113(31.47) |  |
| College and high | 1398(48.34) | 1651(59.84) | 1911(67.19) | 1775(62.60) |  |
| **Marital status, *n* (%)** |  |  |  |  | **0.002** |
| Not married | 1321(39.12) | 1164(33.71) | 1105(32.28) | 1126(32.84) |  |
| Married | 1963(60.88) | 2087(66.29) | 2152(67.72) | 2138(67.16) |  |
| **Annual household income, *n* (%)** |  |  |  |  | **< 0.0001** |
| ＜$20,000 | 790(18.25) | 621(12.82) | 503(10.09) | 615(14.63) |  |
| ≥$20,000 | 2324(81.75) | 2457(87.18) | 2623(89.91) | 2485(85.37) |  |
| **Poverty to income ratio, *n* (%)** |  |  |  |  | **< 0.0001** |
| < 1 | 745(17.73) | 563(12.61) | 424(9.78) | 533(13.60) |  |
| 1-3 | 1393(39.64) | 1317(35.91) | 1234(31.20) | 1218(34.30) |  |
| ≥3 | 852(42.64) | 1066(51.48) | 1324(59.01) | 1193(52.11) |  |
| **Weight status, *n* (%)** |  |  |  |  | **< 0.0001** |
| Normal (BMI＜25) | 867(27.84) | 782(25.27) | 925(32.25) | 875(28.80) |  |
| Overweight (25≤BMI＜30) | 992(28.49) | 1093(33.25) | 1105(32.91) | 1140(34.90) |  |
| Obese (BMI≥30) | 1401(43.67) | 1353(41.48) | 1199(34.84) | 1223(36.31) |  |
| **Alcohol status, *n* (%)** |  |  |  |  | **< 0.0001** |
| Never | 416(11.26) | 419(10.66) | 351(7.73) | 450(12.18) |  |
| Former | 525(13.94) | 481(12.01) | 382(8.72) | 436(10.68) |  |
| Current | 2012(74.80) | 2141(77.33) | 2395(83.56) | 2203(77.14) |  |
| **Smoking status, *n* (%)** |  |  |  |  | **< 0.0001** |
| Never | 1519(48.11) | 1761(56.28) | 1884(59.77) | 1941(59.57) |  |
| Former | 820(24.54) | 786(23.57) | 864(25.73) | 864(25.98) |  |
| Current | 946(27.35) | 706(20.14) | 510(14.50) | 461(14.45) |  |
| **Caffeine intake, mg/day, median (IQR)** | 121.50(44.00,244.50) | 130.00(49.50,252.00) | 130.50(48.00,235.00) | 104.00(25.00,206.50) | **< 0.0001** |
| **Disease measures, mean (SD)** | | | | | |
| PA total time, minutes | 1552.07(76.66) | 1271.63(47.64) | 1114.06(38.09) | 1211.51(51.15) | **< 0.0001** |
| Sitting time, hours | 5.58(0.10) | 5.69(0.10) | 5.79(0.09) | 5.63(0.11) | 0.44 |
| eGFR, mL/min/1.73m^2^ | 92.51(0.69) | 91.31(0.73) | 91.69(0.74) | 91.85(0.83) | 0.53 |
| FPG, mmol/L | 5.99(0.08) | 6.00(0.06) | 5.84(0.04) | 6.02(0.08) | 0.1 |
| Urine creatinine, mg/dL | 131.42(2.67) | 122.84(2.49) | 114.70(1.82) | 119.35(2.26) | **< 0.0001** |
| Uric acid, mg/dL | 5.48(0.03) | 5.51(0.04) | 5.35(0.04) | 5.44(0.05) | **0.01** |
| Hs-CRP, mg/L | 4.32(0.27) | 4.35(0.46) | 3.40(0.36) | 3.64(0.33) | 0.22 |
| Calcium, mg/dL | 9.35(0.02) | 9.38(0.02) | 9.39(0.02) | 9.42(0.01) | **0.01** |
| Blood urea nitrogen, mg/dL | 13.47(0.15) | 13.74(0.13) | 13.98(0.18) | 13.94(0.14) | 0.07 |
| **Dietary measures, median (IQR)** | | | | | |
| Total energy intake, kcal/day | 1879.00(1354.00,2589.00) | 1939.00(1454.00,2573.00) | 1981.00(1493.00,2575.00) | 2040.00(1566.00,2733.00) | **< 0.0001** |
| HEI-2015 total score | 44.11(36.12,53.72) | 47.99(38.74,57.56) | 52.37(43.34,61.49) | 55.49(46.92,64.78) | **< 0.0001** |
| OBS | 17.00(12.00,23.00) | 20.00(14.00,25.00) | 22.00(16.00,27.00) | 23.00(17.00,28.00) | **< 0.0001** |
| CDAI | -1.46(-3.26,0.66) | -0.69(-2.49,1.67) | 0.17(-1.90,2.65) | 0.60(-1.33,3.33) | **< 0.0001** |
| DII | 2.50(1.10,3.53) | 1.94(0.54,3.10) | 1.35(-0.23,2.72) | 0.82(-0.71,2.24) | **< 0.0001** |

SD, Standard deviation; IQR, Interquartile range; BMI, Body mass index; DM, Diabetes Mellitus; IFG, Impaired Fasting Glucose; IGT, Impaired Glucose Tolerance; PA, Physical activity; eGFR, estimated glomerular filtration rate; FPG, Fasting plasma glucose; Hs-CRP: Hypersensitive C-reactive protein; HEI, Healthy eating index; OBS, Oxidative balance score; CDAI, Composite Dietary Antioxidant Index; DII, Dietary Inflammatory Index.

**Supplementary Table 8.** Baseline characteristics of the participants based on the quartiles of the flavan-3-ol intake, weighted.

|  | Flavan-3-ol (Range, mg/day) | | | |  |
| --- | --- | --- | --- | --- | --- |
| Variables | Quartile 1  [0,4.975] | Quartile 2  (4.975,15.63] | Quartile 3  (15.63,157.757] | Quartile 4  (157.757,6724.88] | *P*-value |
| **OAB, *n* (%)** |  |  |  |  | 0.07 |
| No | 2503(81.12) | 2565(84.82) | 2563(83.10) | 2592(83.71) |  |
| Yes | 764(18.88) | 700(15.18) | 702(16.90) | 674(16.29) |  |
| **Demographic** | | | | | |
| **Age, *n* (%)** |  |  |  |  | **< 0.0001** |
| 20-41 | 1298(46.82) | 1104(41.62) | 1034(36.66) | 1014(35.56) |  |
| 42-61 | 1043(32.91) | 1099(35.60) | 1160(38.70) | 1207(41.98) |  |
| 62-80 | 926(20.28) | 1062(22.78) | 1071(24.64) | 1045(22.46) |  |
| **Sex, *n* (%)** |  |  |  |  | 0.15 |
| Male | 1582(47.33) | 1624(50.20) | 1572(48.28) | 1528(45.70) |  |
| Female | 1685(52.67) | 1641(49.80) | 1693(51.72) | 1738(54.30) |  |
| **Race, *n* (%)** |  |  |  |  | **< 0.0001** |
| Non-Hispanic White | 1409(63.39) | 1473(67.69) | 1403(65.71) | 1723(72.71) |  |
| Mexican American | 564(10.24) | 582(10.09) | 553(8.95) | 341(4.97) |  |
| Non-Hispanic Black | 793(14.59) | 643(10.88) | 634(10.83) | 615(9.26) |  |
| Non-Hispanic Asian | 57(1.09) | 98(1.80) | 130(2.35) | 195(2.85) |  |
| Other/Multi-Racial | 444(10.68) | 469(9.53) | 545(12.16) | 392(10.21) |  |
| **Education level, *n* (%)** |  |  |  |  | **< 0.0001** |
| Less than high school | 425(6.76) | 370(5.24) | 326(4.62) | 194(3.05) |  |
| High school | 1463(43.28) | 1285(37.84) | 1103(29.28) | 1148(33.04) |  |
| College and high | 1377(49.96) | 1607(56.93) | 1831(66.10) | 1920(63.91) |  |
| **Marital status, *n* (%)** |  |  |  |  | 0.17 |
| Not married | 1285(36.92) | 1169(34.43) | 1149(33.66) | 1113(33.22) |  |
| Married | 1981(63.08) | 2094(65.57) | 2115(66.34) | 2150(66.78) |  |
| **Annual household income, *n* (%)** |  |  |  |  | **< 0.0001** |
| ＜$20,000 | 770(18.06) | 636(13.88) | 582(11.88) | 541(12.04) |  |
| ≥$20,000 | 2352(81.94) | 2472(86.12) | 2485(88.12) | 2580(87.96) |  |
| **Poverty to income ratio, *n* (%)** |  |  |  |  | **< 0.0001** |
| < 1 | 732(18.86) | 562(12.83) | 542(11.99) | 429(10.34) |  |
| 1-3 | 1407(38.87) | 1363(37.77) | 1199(31.88) | 1193(32.95) |  |
| ≥3 | 851(42.26) | 1049(49.41) | 1185(56.13) | 1350(56.71) |  |
| **Weight status, *n* (%)** |  |  |  |  | **< 0.0001** |
| Normal (BMI＜25) | 795(24.92) | 859(28.03) | 912(30.77) | 883(30.06) |  |
| Overweight (25≤BMI＜30) | 959(28.67) | 1108(33.65) | 1147(33.48) | 1116(33.29) |  |
| Obese (BMI≥30) | 1483(46.40) | 1263(38.32) | 1184(35.75) | 1246(36.65) |  |
| **Alcohol status, *n* (%)** |  |  |  |  | **0.01** |
| Never | 405(9.96) | 375(10.33) | 431(10.68) | 425(10.38) |  |
| Former | 543(14.17) | 440(10.82) | 375(8.86) | 466(11.42) |  |
| Current | 2059(75.87) | 2244(78.85) | 2247(80.46) | 2201(78.20) |  |
| **Smoking status, *n* (%)** |  |  |  |  | **< 0.0001** |
| Never | 1570(48.66) | 1735(55.14) | 1956(61.74) | 1844(57.42) |  |
| Former | 798(24.25) | 870(25.03) | 833(24.80) | 833(25.54) |  |
| Current | 899(27.09) | 660(19.83) | 475(13.47) | 589(17.04) |  |
| **Caffeine intake, mg/day, median (IQR)** | 103.00(26.00,210.50) | 103.50(21.50,221.00) | 102.00(24.50,220.00) | 163.00(84.50,282.50) | **< 0.0001** |
| **Disease measures, mean (SD)** | | | | | |
| PA total time, minutes | 1469.31(75.72) | 1277.25(46.84) | 1187.86(47.20) | 1227.25(48.07) | **0.01** |
| Sitting time, hours | 5.64(0.11) | 5.61(0.07) | 5.83(0.13) | 5.63(0.09) | 0.35 |
| eGFR, mL/min/1.73m^2^ | 93.57(0.78) | 92.29(0.67) | 91.51(0.86) | 90.31(0.67) | **0.01** |
| FPG, mmol/L | 6.02(0.06) | 5.88(0.04) | 6.02(0.09) | 5.91(0.06) | 0.11 |
| Urine creatinine, mg/dL | 131.79(2.33) | 121.72(2.38) | 117.85(2.30) | 117.87(2.54) | **< 0.001** |
| Uric acid, mg/dL | 5.49(0.05) | 5.50(0.05) | 5.34(0.04) | 5.45(0.04) | **0.03** |
| Hs-CRP, mg/L | 5.19(0.77) | 3.60(0.16) | 3.31(0.15) | 3.89(0.36) | 0.15 |
| Calcium, mg/dL | 9.37(0.02) | 9.42(0.01) | 9.38(0.02) | 9.37(0.02) | **0.01** |
| Blood urea nitrogen, mg/dL | 13.54(0.17) | 13.66(0.16) | 14.30(0.15) | 13.64(0.14) | **< 0.001** |
| **Dietary measures, median (IQR)** | | | | | |
| Total energy intake, kcal/day | 1732.00(1251.00,2356.00) | 1978.00(1476.00,2608.00) | 2158.00(1631.00,2786.00) | 1997.00(1520.00,2664.00) | **< 0.0001** |
| HEI-2015 total score | 43.75(35.87,52.42) | 50.43(41.77,59.44) | 54.75(45.38,64.09) | 51.22(41.32,60.87) | **< 0.0001** |
| OBS | 17.00(11.00,22.00) | 20.00(15.00,26.00) | 23.00(17.00,28.00) | 21.00(15.00,27.00) | **< 0.0001** |
| CDAI | -1.43(-3.24,0.82) | -0.38(-2.39,1.84) | 0.35(-1.45,2.82) | -0.12(-2.15,2.40) | **< 0.0001** |
| DII | 2.55(1.21,3.55) | 1.71(0.28,2.84) | 0.94(-0.55,2.38) | 1.52(-0.03,2.95) | **< 0.0001** |

SD, Standard deviation; IQR, Interquartile range; BMI, Body mass index; DM, Diabetes Mellitus; IFG, Impaired Fasting Glucose; IGT, Impaired Glucose Tolerance; PA, Physical activity; eGFR, estimated glomerular filtration rate; FPG, Fasting plasma glucose; Hs-CRP: Hypersensitive C-reactive protein; HEI, Healthy eating index; OBS, Oxidative balance score; CDAI, Composite Dietary Antioxidant Index; DII, Dietary Inflammatory Index.

## Supplementary Figures


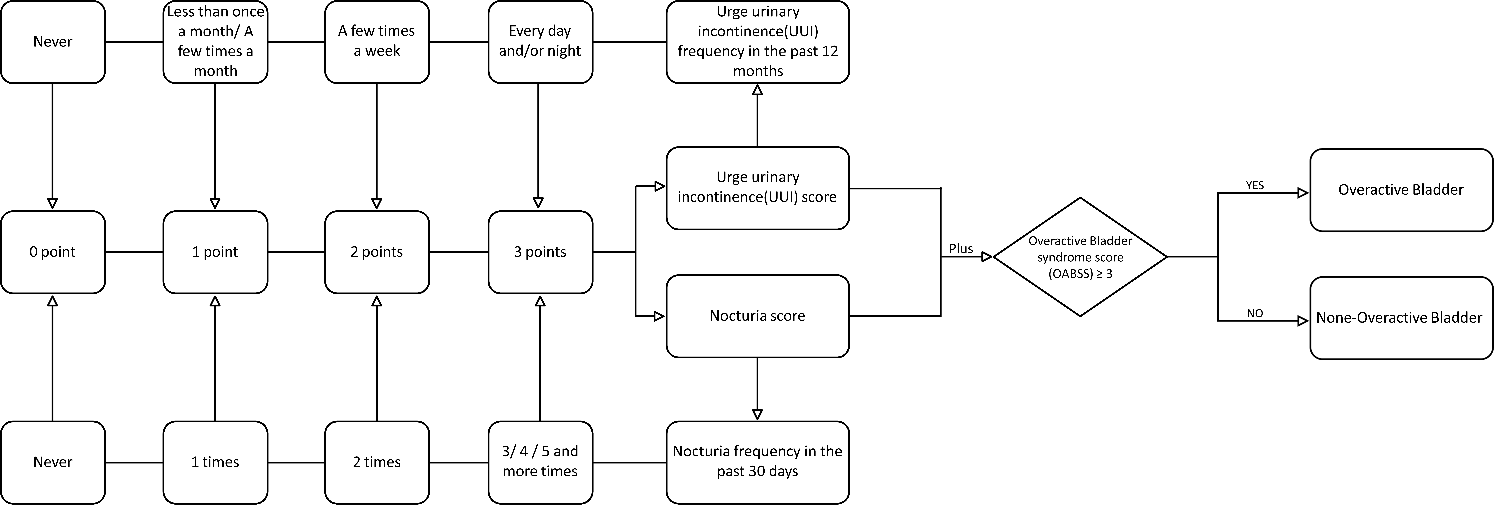


**Supplementary Figure 1.** Flow diagram of the overactive bladder diagnosis based on overactive bladder syndrome score.


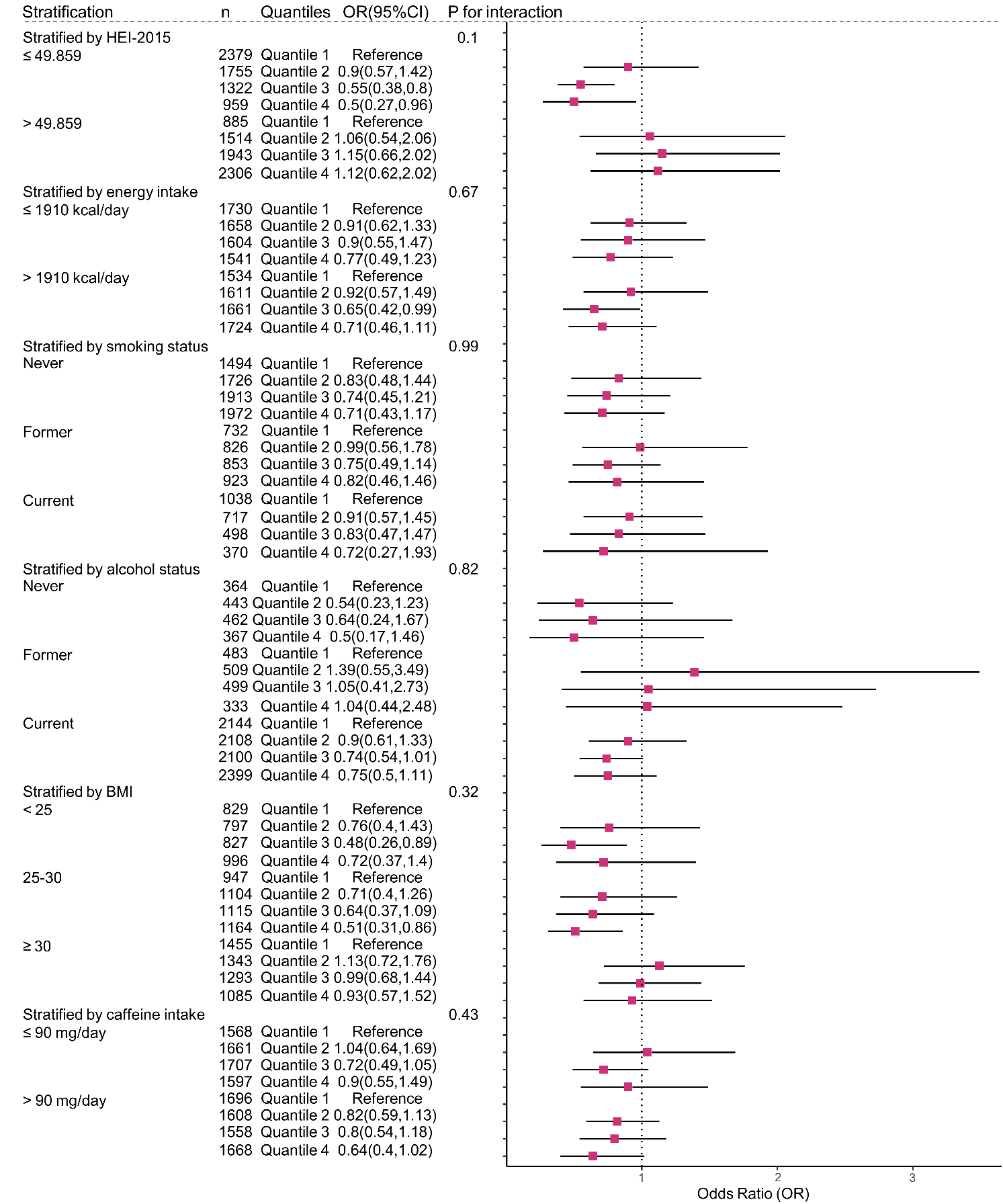
**Supplementary Figure 2.** The weighted stratified and interaction analysis of association between anthocyanidin intake and covariates. All of the confounding factors that were used in the model 4 were adjusted.


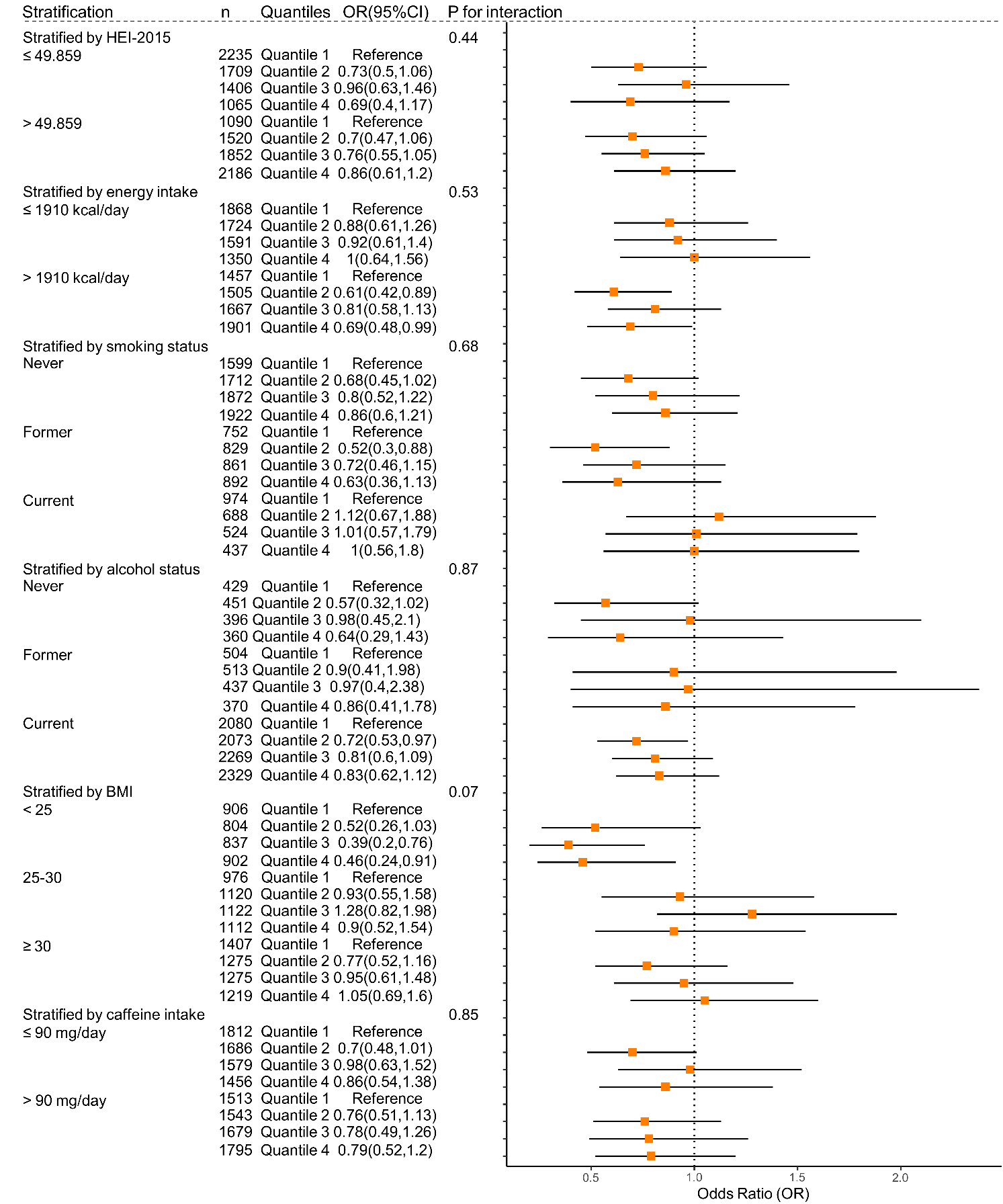
**Supplementary Figure 3.** The weighted stratified and interaction analysis of association between flavone intake and covariates. All of the confounding factors that were used in the model 4 were adjusted.
